# Regional species turnover and local host and forest stand characteristics jointly structure early foliar mycobiomes of Scots pine seedlings

**DOI:** 10.1093/femsec/fiag114

**Published:** 2026-10-05

**Authors:** Werna Wahlman, Sannakajsa Velmala, Suvi Sutela, Eeva Terhonen

**Affiliations:** Forest Health and Biodiversity, Natural Resources Institute Finland (Luke), Latokartanonkaari 9, 00790 Helsinki, Finland; Forest Health and Biodiversity, Natural Resources Institute Finland (Luke), Latokartanonkaari 9, 00790 Helsinki, Finland; Forest Health and Biodiversity, Natural Resources Institute Finland (Luke), Latokartanonkaari 9, 00790 Helsinki, Finland; Forest Health and Biodiversity, Natural Resources Institute Finland (Luke), Latokartanonkaari 9, 00790 Helsinki, Finland

**Keywords:** foliar mycobiome, *Pinus sylvestris*, DNA metabarcoding, fungal diversity, community composition, forest regeneration

## Abstract

The foliar mycobiome plays an important role in tree health and stress resilience, yet factors shaping foliar fungal communities in boreal conifer seedlings remain poorly understood. We investigated how geographic region, seedling origin (planted vs. naturally regenerated), needle nitrogen status, and local stand structure influence needle-associated fungal communities of Scots pine (*Pinus sylvestris*) seedlings. Needle samples were collected from seedlings without visible disease symptoms across 23 forest stands in southern Finland. Fungal communities were characterized using Illumina ITS2 amplicon sequencing and clustered into operational taxonomical units (OTUs). Alpha diversity was analyzed via linear mixed models (LMMs), and community composition via PERMANOVA and NMDS using Bray–Curtis dissimilarity. Geographic region was the strongest driver of fungal community composition among stands, whereas within stands, community composition was primarily associated with seedling height and birch abundance. Alpha diversity was higher in the Southeastern region compared to the Southwest, and tended to be lower in planted than naturally regenerated seedlings. Moreover, diversity decreased with higher proportions of birch and spruce but increased with higher needle C:N ratios. These findings suggest that early foliar mycobiomes reflect regional conditions and local forest structure, highlighting potential forest management impacts on fungal communities.

## Teaser

Early foliar mycobiomes of Scots pine seedlings are structured by regional species turnover and local host and forest stand characteristics, while potentially pathogenic fungi commonly occur in asymptomatic needles.

## Introduction

The early seedling stage represents a critical phase of forest regeneration, during which seedlings are particularly vulnerable to environmental stress, pathogen infection, and mortality (Johnson et al. 2011, Harrison and LaForgia 2019). Disturbances at this stage can have lasting effects on future forest development by reducing regeneration success, increasing the need for costly replanting, and delaying the establishment of productive forest stands. Successful early establishment is particularly important in Northern Europe, where forestry relies on clear-cutting followed by artificial regeneration through planting or seeding (Nilsson et al. 2010). At the same time, climate change is posing new challenges, such as fluctuating weather conditions, more intense drought and emerging pests and pathogens (Seidl et al. 2017, Forzieri et al. 2021, Rantanen et al. 2022), which may hinder forest regeneration.

Scots pine (*Pinus sylvestris* L.) is the most widely distributed conifer species in Europe and has considerable ecological and economic importance across boreal and temperate regions (Brichta et al. 2023). Its needles host diverse fungal communities, including a wide range of endophytic and epiphytic taxa (Terhonen et al. 2011, Blumenstein et al. 2021a, Carvajal-Arias et al. 2026). These fungi exhibit diverse ecological strategies, functioning as mutualists, commensals, latent pathogens or saprotrophs, with roles that may shift depending on host condition and environmental context (Arnold 2007, Porras-Alfaro and Bayman 2011). Many persist asymptomatically as endophytes and can enhance host resilience by improving drought tolerance, altering plant physiology, increasing biomass, reducing herbivory, or suppressing pathogens through competition and antibiosis (Rodriguez et al. 2009, Porras-Alfaro and Bayman 2011, Sumarah et al. 2011, 2015, Ullah et al. 2025). However, interactions between endophytes and pathogens are not always antagonistic, and facilitative effects may also occur (Ridout and Newcombe 2015). Rather than acting independently, foliar fungi form complex communities, where overall composition and balance may affect host stress tolerance and disease susceptibility.

Previous studies have shown that fungal species richness and community composition vary across broad geographic gradients, with richness generally decreasing from south to north (Terhonen et al. 2011, Millberg et al. 2015, Agan et al. 2021). Locally, factors such as air movement, humidity and “founder effects” from the surrounding vegetation may create distinct fungal communities even between individual trees within the same forest patch (Taudière et al. 2018, Seabloom et al. 2019, Schönrogge et al. 2022).

At the host level, foliar fungal communities are shaped by both species identity and intraspecific genetic variation (Hantsch et al. 2013, Kembel and Mueller 2014, Schönrogge et al. 2022), as well as by host developmental traits. Mature trees typically host higher foliar endophyte densities than seedlings, although seedlings may sometimes show higher diversity (Oono et al. 2015). Older needles tend to harbor more diverse and compositionally distinct fungal communities, likely due to longer exposure to fungal colonization and age-related changes in needle physiology (Helander et al. 1994, Hata et al. 1998, Deckert and Peterson 2000, Millberg et al. 2015b, Agan et al. 2021). While nursery practices and cultivation system can have a profound effect on root-associated fungal communities (Menkis et al. 2005, Okorski et al. 2019), similar effects have not been observed for foliar fungi (López-Fernández 2023).

In boreal and temperate forest systems, nitrogen is a key determinant of plant physiology and associated microbial communities (Meng et al. 2022). Variation in host nutrient status may shape foliar fungal diversity and composition through resource competition among taxa and by influencing the balance between endophytic and pathogenic lifestyles (Oliva et al. 2021, Simha et al. 2022). For example, endophytes with broad, overlapping metabolic profiles can suppress pathogens through competitive exclusion (Blumenstein et al. 2015).

Despite growing research on the Scots pine mycobiome, the factors shaping foliar fungal communities during early seedling establishment remains poorly understood. Given the potential roles of foliar fungi in plant stress tolerance and disease dynamics (Busby et al. 2016, Terhonen et al. 2019), understanding mycobiome assembly during the critical seedling stage could provide key insights into the microbial processes shaping forest regeneration under changing environmental conditions. This study focuses on regenerating forest stands established under standard forestry practices in privately owned forests in southern Finland. This allows us to examine fungal community assembly under operational forest management conditions rather than experimental settings.

The aim of this study was to assess how regional context, local tree species structure, forest regeneration pathway, and host C:N ratio shape needle–associated fungal communities in asymptomatic Scots pine seedlings. Specifically, we examined their effects on mycobiome diversity, community composition, and functional structure. Our main research question was:

What shapes the taxonomic and functional structure of Scots pine seedling foliar mycobiomes across regional, stand-level, and host-related conditions?

We hypothesized that:

1. H1 – Geographic region structures foliar fungal community composition and diversity, with regional differences in environmental conditions and species pools expected to contribute to this variation.
2. H2 – Surrounding tree species composition shapes the taxonomic and functional structure of Scots pine foliar fungal communities.
3. H3 – Foliar fungal communities in the most recent annual shoots do not differ significantly between naturally regenerated and nursery–planted seedlings, given similar needle age and exposure time.
4. H4 – Foliar fungal diversity and composition vary with needle C:N ratio, reflecting differences in host nitrogen status.

## Materials and methods

### Site description and field sampling

Field work was done during spring (April–May) 2025, before seedling growth had started, in two different regions: Southwestern Finland archipelago and Southeastern Finland inland area (Fig. 1). In addition, one seedling stand was in Kanta-Häme, southern Finland. All measured seedling stands were on private land and had been regenerated between 2018 and 2023, with a median regeneration year of 2021. One to five study plots were established in each stand, depending on the stand size and species composition. Stand composition ranged from pine-dominated to mixed stands including Scots pine, Norway spruce, and birch. Each site had typically both planted and naturally regenerated Scots pine and Norway spruce, whereas birch was exclusively naturally regenerated. While most seedling stands were classified as mesic or sub-xeric heath forests, study plots encompassed a wide range of local growing conditions, from herb-rich to xeric heath microsites.

**Figure 1:**
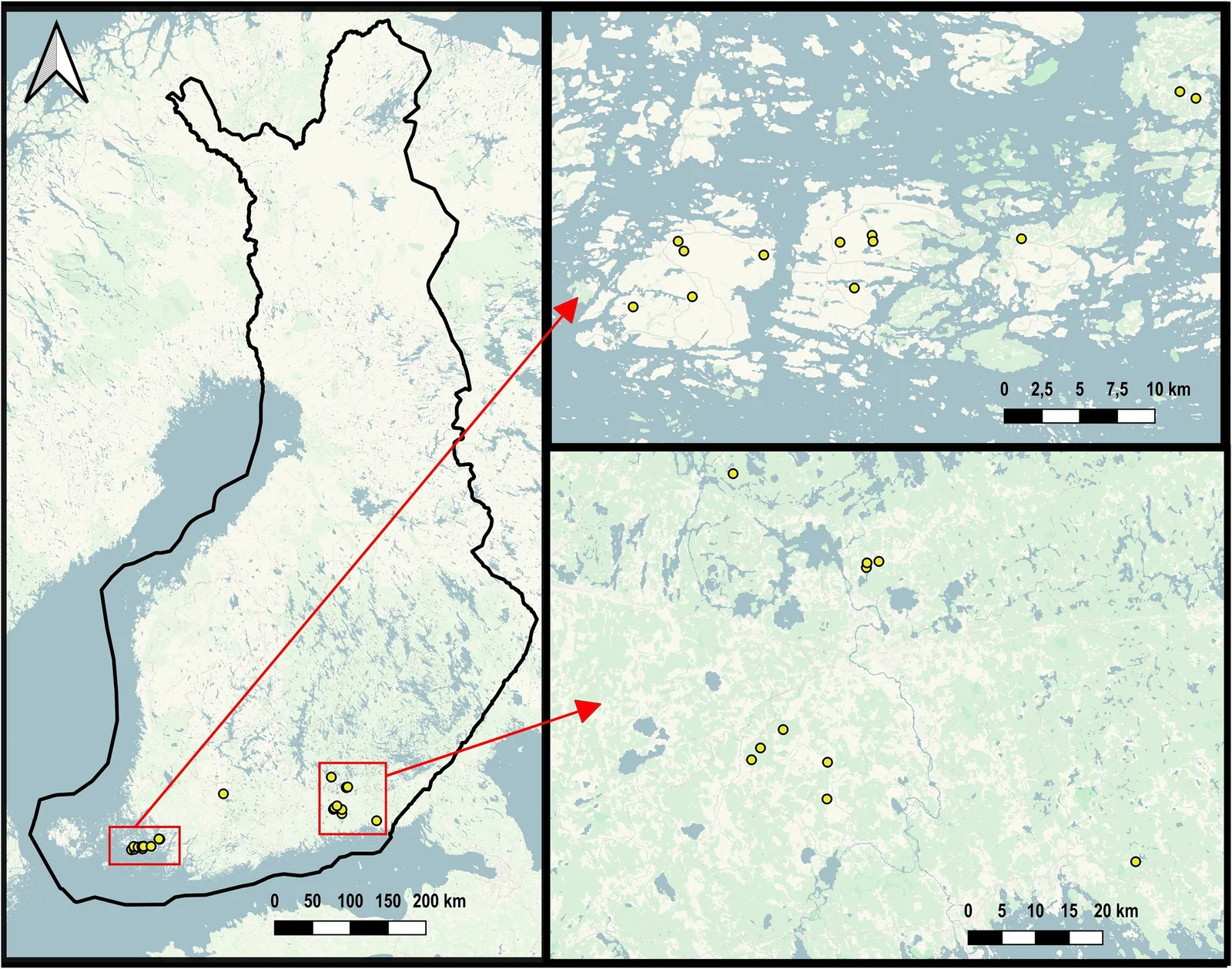
Locations of sampled Scots pine seedling stands in southern Finland. The left panel shows the study regions within Finland, and the right panels show the distribution of individual study stands in Southwestern and Southeastern Finland. Points indicate sampled stands.

In total, three plots from Kanta-Häme, 44 plots from Southwest Finland and 21 plots from Southeastern Finland were measured. Sampling plot radius was 3.99 m, totaling surface area of 50 m^2^. In each plot, height (to the nearest 1 cm), annual height growth from the last 2 years (to the nearest 1 cm), and stem base diameter (to the nearest 0.5 cm) were measured from all Scots pine and Norway spruce seedlings. Diameter was measured with vernier caliper and height with measuring rod. Both naturally regenerated and planted seedlings were measured, and the origin was recorded. Additionally, due to high abundance at some sites, birch seedlings were not measured individually; instead, only the total count of birch seedlings ≥15 cm in height was recorded from each plot. More detailed information on sampling effort and stand characteristics is provided in Supplementary Table S1.

Needle samples were collected from three Scots pine seedlings per plot. Across all seedling stands, 3–15 needle samples were taken depending on the number of study plots (following stand size) and species composition (mixture vs. monoculture) of the stand. In total, 193 needle samples were collected. Needle samples were taken from the latest annual shoot of Scots pine seedlings without visible foliar disease symptoms. Although all sampled seedlings appeared free of obvious disease symptoms or signs of stress, they occurred across a range of stand and microsite conditions and exhibited natural variation in height and growth characteristics. The needles were collected into their own zip-lock bags, stored in a cooler in the field, and put in a −20°C freezer maximum 8 h after collection before further processing.

### Scots pine needle processing

Frozen needles were ground to a fine powder under liquid nitrogen using a mortar and pestle. The number of individual needles per sample varied; therefore, downstream analyses were standardized by sample mass. Total genomic DNA was extracted from 100 mg of the resulting powder using the DNeasy Plant Pro Kit (Qiagen Ltd) on a QIAcube automated platform, following the manufacturer’s instructions. The remaining plant material was used for nutrient analyses.

DNA purity and concentration were assessed using a NanoDrop One spectrophotometer (Thermo Fisher Scientific), and double–stranded DNA was quantified using the Qubit dsDNA High Sensitivity Assay Kit (Thermo Fisher Scientific). DNA quality metrics for all samples are available in the associated Zenodo repository. DNA concentrations were normalized based on Qubit measurements and sent to Macrogen Europe (Amsterdam, The Netherlands), where libraries were prepared and the fungal ITS2 region was amplified using the primer pair ITS3 (5′-GCATCGATGAAGAACGCAGC-3′) (White et al. 1990) and ITS4 (5′-TCCTCCGCTTATTGATATGC-3′) (White et al. 1990). Sequencing was carried out on the Illumina MiSeq i100 platform using 2 × 300 bp chemistry. Extraction blanks and PCR negative controls were included throughout laboratory processing and sequenced alongside biological samples.

For C:N analysis, homogenized needle samples were oven-dried in paper bags at +40°C. From dry material total carbon and nitrogen concentrations were analyzed using a LECO CN928 elemental analyzer.

### Bioinformatics

Raw data generated in this research are available in the European Nucleotide Archive at EMBL-EBI under accession number PRJEB114804. Raw paired-end ITS2 amplicon reads were processed using the DADA2 pipeline (Callahan et al. 2016) in RStudio (v4.6.0). Primer sequences (ITS3/ITS4) were removed from raw reads using Cutadapt (Martin 2011), using the following parameters: two possible primer occurrences per read (-n 2), minimum overlap of 10 bp (-o 10) and removal of reads containing ambiguous bases (–max -n 0). After primer removal, read quality remained high across full lengths, and no additional trimming or truncation was applied.

Reads containing ambiguous bases or >2 expected errors were discarded, and PhiX sequences were removed. Error models were learned separately for forward and reverse reads (2 × 10⁸ bases), and amplicon sequence variants (ASVs) were inferred using the DADA algorithm. Paired reads were merged with a minimum overlap of 12 bp and no mismatches allowed. Chimeric sequences were removed using a stringent threshold (minFoldParentOverAbundance = 2) to prevent losing genuine low abundance ITS variants.

Taxonomy was assigned using the DADA2 naive Bayesian classifier against the UNITE database (Abarenkov et al. 2025) version 10.0. Only fungal ASVs were retained, after which selected taxa (e.g. *Hormonema, Diplodia, Alternaria, Lophodermium*,and *Fusarium*) were further curated using BLASTn against GenBank (Sayers et al. 2011) to refine species-level identifications. Species-level identifications were accepted only when high-scoring BLAST matches showed consistent ≥99%–100% identity to a single species; taxa with ambiguous species-level matches, including *Fusarium* and *Alternaria*, were conservatively retained at the genus level. Additional details are provided in the Supplementary Methods.

Negative controls were processed through the same DADA2 pipeline as the biological samples and were inspected for potential contamination. No ASVs were shared between the two negative controls, and no evidence of systematic contamination was detected. Consequently, no additional contaminant filtering was applied, and control samples were removed from the dataset prior to downstream analyses.

Although ASVs capture biologically meaningful sequence variation within fungal species, our focus was on community patterns at an approximate species level rather than on intraspecific sequence variation. Therefore, to reduce potential inflation of richness due to intragenomic ITS variation, ASVs were clustered into operational taxonomic units (OTUs) at 97% sequence similarity using VSEARCH v2.28.1 (Rognes et al. 2016). Recent studies have shown that ASV- and OTU-based analyses generally recover similar ecological patterns, while OTUs provide a more conservative approximation of species-level richness in fungal metabarcoding datasets (Tedersoo et al. 2022, Kyaschenko et al. 2026).

Clustering was performed using the cluster_size algorithm, which orders sequences by decreasing abundance and retains the most abundant sequence as the cluster centroid. Singleton and rare OTUs (<0.01% relative abundance) were removed prior to downstream analyses.

Additional details of sequence processing and taxonomic curation are provided in Supplementary Methods file, and all ASV and OTU tables can be downloaded from (https://doi.org/10.5281/zenodo.20589402).

### Statistical analysis

Prior to statistical modeling, the dataset was filtered to remove potential confounding factors. Of the 193 sampled Scots pine seedlings, six seedlings from Kanta-Häme were excluded because the area contained only a few plots with exclusively planted seedlings, preventing the effects of geographic region and seedling origin from being disentangled. Consequently, all statistical analyses were restricted to Southwest and Southeast Finland, where both planted and naturally regenerated seedlings were well represented, resulting in a dataset of 187 seedlings. Models including needle C:N ratio were fitted using 186 observations because C:N measurements were unavailable for one seedling.

OTU tables were normalized using the GMPR (geometric mean of pairwise ratios) method, which accounts for differences in library size while preserving relative abundance structure in sparse microbiome data (Chen et al. 2018). To assess the robustness of results to sequencing depth, analyses were repeated using rarefied OTU tables. One sample with extremely low sequencing depth (<30 000 reads) was excluded prior to rarefaction to avoid unnecessary loss of information. The remaining samples were rarefied to 30 000 reads per sample. Rarefaction curves are provided in Supplementary Fig. S10.

#### Functional annotation

Functional guilds and trophic modes were assigned using FUNGuild (Nguyen et al. 2016). Here, only assignments with “Probable” and “Highly probable” confidence were retained, and others were marked as unassigned.

#### Exploratory analyses

Taxonomic composition was summarized using relative abundance plots of dominant fungal families and genera. Functional guild composition was similarly summarized to describe broad trophic patterns across regions, seedling origins, and stand types. Associations with stand composition were explored using scatterplots and Pearson correlations based on GMPR–normalized OTU abundances aggregated by trophic group. These analyses are descriptive and intended to illustrate general patterns, therefore, they were not used for hypothesis testing. Because tree species proportions are compositional (summing to one within plots), correlations reflect relative stand–composition gradients rather than independent effects. Formal statistical inference focused on diversity indices and community composition analyses described below.

#### Alpha diversity

Alpha diversity (Shannon and Simpson indices) was calculated using the estimate richness function in the phyloseq package (McMurdie and Holmes 2017). Linear mixed-effects models were fitted using the lme4-package (Bates et al. 2015), with stand included as a random intercept (1 | Stand). An initially tested nested structure (plot within stand) was simplified, because the plot-level variance component was estimated as zero (singular fit), indicating that stand accounted for all higher-level hierarchical variation.

Based on data exploration of the full dataset, we selected eight ecologically meaningful covariates to describe site conditions, stand structure and host traits (Table 1). Multicollinearity among predictors was assessed using Pearson correlations and variance inflation factors (VIF). All predictors showed low pairwise correlations and VIF values <2, indicating no multicollinearity issues. Although tree species proportions are constrained within sampling plots, birch and spruce proportions showed low collinearity and were therefore included simultaneously as covariates. Site type was simplified into two categories for analysis: herb-rich and mesic heath sites were combined into a single mesic category, whereas sub-xeric and xeric heath sites were combined into a xeric category.

**Table 1:** Covariates used in statistical analyses of fungal diversity and community composition.

Covariate	Type	Description and ecological rationale
**Geographic region (Area)**	Categorical (Southwestern Finland archipelago and Sout-Eastern Finland inland area)	Represents an east–west biogeographic gradient as well as the continental–maritime climatic transition. Used to account for major regional environmental differences
**Seedling origin (Origin)**	Categorical (Planted/naturally regenerated)	Captures differences in establishment history and potential nursery effects.
**Needle C:N ratio (NeedleCN)**	Continuous	Reflects variation in needle C:N stoichiometry and primarily serves as a proxy for needle nitrogen concentration.
**Spruce proportion within sampling plot (PropSpruce)**	Continuous (0–1)	Relative abundance of *Picea abies* within the sampling plot; describes stand species composition.
**Birch proportion within sampling plot (PropBirch)**	Continuous (0–1)	Relative abundance of *Betula* spp. in the plot; complements spruce proportion. Also affects microclimatic conditions when abundant.
**Regeneration year**	Continuous	Year of stand establishment, used as a proxy for seedling age and stand developmental stage.
**Site type**	Categorical (Mesic/xeric)	Describes site fertility conditions known to affect fungal and tree community structure.
**Seedling height, cm**	Continuous	Proxy for individual vigor, growth history, and microenvironmental variation among seedlings.

For both Shannon and Simpson diversity, the initial full model included all eight covariates. We did not use stepwise model selection or *P*-value filtering, because these methods may remove variables that are ecologically relevant even if they are not statistically significant (Tedersoo et al. 2022). Instead, model selection was based on semi partial *R*^2^ values, retaining all predictors explaining ≥1% of the variance. This approach emphasizes effect size and ecological relevance over arbitrary significance thresholds, while maintaining a transparent and reproducible selection criterion. Semi-partial *R*² values and confidence intervals for all candidate predictors for both models are provided in supplementary material (Tables S2 and S4).

An interaction term (Origin × NeedleCN) was tested but did not improve model fit and was excluded. The final model was:


\begin{eqnarray*}
&&{\mathop{\mathrm{Re}}\nolimits} {\rm sponse}\ {\mathop{\mathrm{var}}} {\rm iable}\ \left( {\mathrm{Shannon}/\rm Simpson} \right) \sim \mathrm{Area}\ + \ \mathrm{Origin}\\&& + \ \mathrm{NeedleCN}\ + \mathrm{PropSpruce}\ + \mathrm{PropBirch}\ + \ \left( {1\ |\ \rm Stand} \right).
\end{eqnarray*}

Model assumptions were evaluated using standard diagnostic plots (residuals vs. fitted values, Q–Q plots, residual distributions and VIF-values; Supplementary Figs S4–S6), which indicated no major violations of model fit. Although mild heteroscedasticity was observed, it was not considered sufficient to affect model interpretation.

To compare needle nutrient status between naturally regenerated and planted seedlings, mean needle C, N, and C:N ratios were calculated for each origin group (naturally regenerated and planted), and differences were tested using two–sample *t*–tests.

#### Beta diversity

Community composition was analyzed using Bray-Curtis dissimilarities calculated from GMPR-normalized OTU abundances. Non-metric multidimensional scaling (NMDS) was conducted using the metaMDS function in the vegan package (Oksanen et al. 2026), and 95% confidence ellipses were added for visualization.

Permutational multivariate analysis of variance (PERMANOVA), implemented with the adonis2 function in the vegan package, was used to statistically test the effects of environmental and host factors on community composition. Homogeneity of multivariate dispersions was assessed using betadisper function. Significant dispersion differences were detected for origin, indicating that community differences between naturally regenerated and planted seedlings could be driven by unequal variance rather than true differences in centroids. As this makes PERMANOVA results for origin potentially unreliable, origin was left out.

In our dataset, area and stand are nested factors: stands belong to specific areas and samples within a stand share environmental conditions. Because of this structure, a single PERMANOVA model cannot simultaneously test both broad scale regional effects and fine scale within stand effects without compromising the interpretation of the results. Therefore, two complementary models were fitted to distinguish sources of variation. The first model included area as a fixed effect without permutation restriction, testing broad-scale differences. The second model excluded area and restricted permutations within stand (strata = Stand), testing local effects. Together, the two models separately capture large–scale regional effects and fine–scale within–stand drivers of community composition. Final models retained predictors explaining ≥1% of variation (*R*² ≥ 0.01). *R*² values for all candidate predictors of both models are provided in Supplementary Tables S6 and S8.

#### Differential abundance analysis

Differential abundance was tested using the ancombc2 function in the ANCOMBC package (Lin and Peddada 2024), on raw (non-normalized) OTU counts, as the method internally accounts for compositional bias and differences in library size. The analysis was conducted at the OTU level with the default settings. Results were summarized at the genus level by aggregating significant taxa and reporting median log fold changes.

## Results

### Seedling characteristics

Sampled seedlings ranged from 12 to 208 cm in height (mean = 68 cm, median = 59 cm) and the most recent annual shoot growth (2024) ranged from 5 to 74 cm (mean = 27 cm, median = 24 cm). Needle nitrogen concentrations ranged from 0.70% to 2.71% (mean = median = 1.43%), whereas carbon concentrations showed considerably less variation (45.79%–49.79%; mean = 47.93%, median = 48.0%). Needle C:N ratios ranged from 17.3 to 69.0 and were driven primarily by variation in nitrogen concentration, as carbon concentrations remained relatively constant among seedlings. Nitrogen concentrations were slightly higher in planted seedlings than in naturally regenerated seedlings (1.44% vs. 1.38%). Conversely, needle C:N ratios were slightly lower in planted seedlings (34.19) than in naturally regenerated seedlings (36.17). However, neither difference was statistically significant.

### Sequencing output and data processing

Illumina ITS2 sequencing generated 20 769 ASVs and 15.7 million reads across all Scots pine needle samples. After removing non-fungal taxa, 17 678 fungal ASVs and 96.8% of reads remained. Sequencing depth varied substantially among samples from 18 000 to 473 000 reads, with a median of ~68 000. ASV abundances were highly skewed: most ASVs were rare (median 38 reads), while a few taxa were strongly dominant (maximum 4.85 million reads).

Clustering with VSEARCH resulted in 2286 OTUs, of which 2125 remained after removal of extremely rare OTUs. This filtered dataset consisted of 193 sample libraries with a median library size of 67 864 reads (range 18 337–473 369).

These values include all samples processed through the bioinformatic pipeline. Samples from Kanta–Häme were excluded from all downstream analyses due to confounding between region and seedling origin and the limited sample size. After exclusion of Kanta-Häme, the final dataset comprised 187 sample libraries, with a median library size of 68 099 reads (range 18 337–473 369) and 2059 OTUs retained for statistical analyses.

### Mycobiome composition

At the phylum level, fungal communities were dominated by *Ascomycota*, accounting for ~64%–69% of reads across regions, followed by *Basidiomycota* (∼11%–20%). All other phyla contributed only minor proportions (<10% combined), and ~7%–8% of reads remained unclassified.

At the family level, fungal communities were dominated by *Dothideaceae*, which had substantially higher relative abundance than any other family across both regions (mean 5%–6%). Most of the dominant families showed relatively similar abundances between regions. In contrast, some less abundant families showed region-specific patterns despite their relatively low overall abundance across entire dataset. For example, *Mniaeciaceae* exhibited a pronounced regional difference, with higher relative abundance in Southeast Finland (∼1.1%) compared to Southwest Finland (∼0.05%). At the genus level, a similar overall structure was observed, with a small number of genera accounting for a substantial proportion of reads alongside a large number of low-abundance taxa. The most abundant genus was *Sydowia*, which consistently dominated communities across both regions (mean 5%–6%).

In addition to regional patterns, substantial variation in community composition was observed among individual stands within both regions (Figs 2 and S1). Some stands were characterized by strong dominance of a single taxon, while others showed more even distributions among multiple families and genera, indicating pronounced heterogeneity in foliar fungal communities at the local scale.

**Figure 2:**
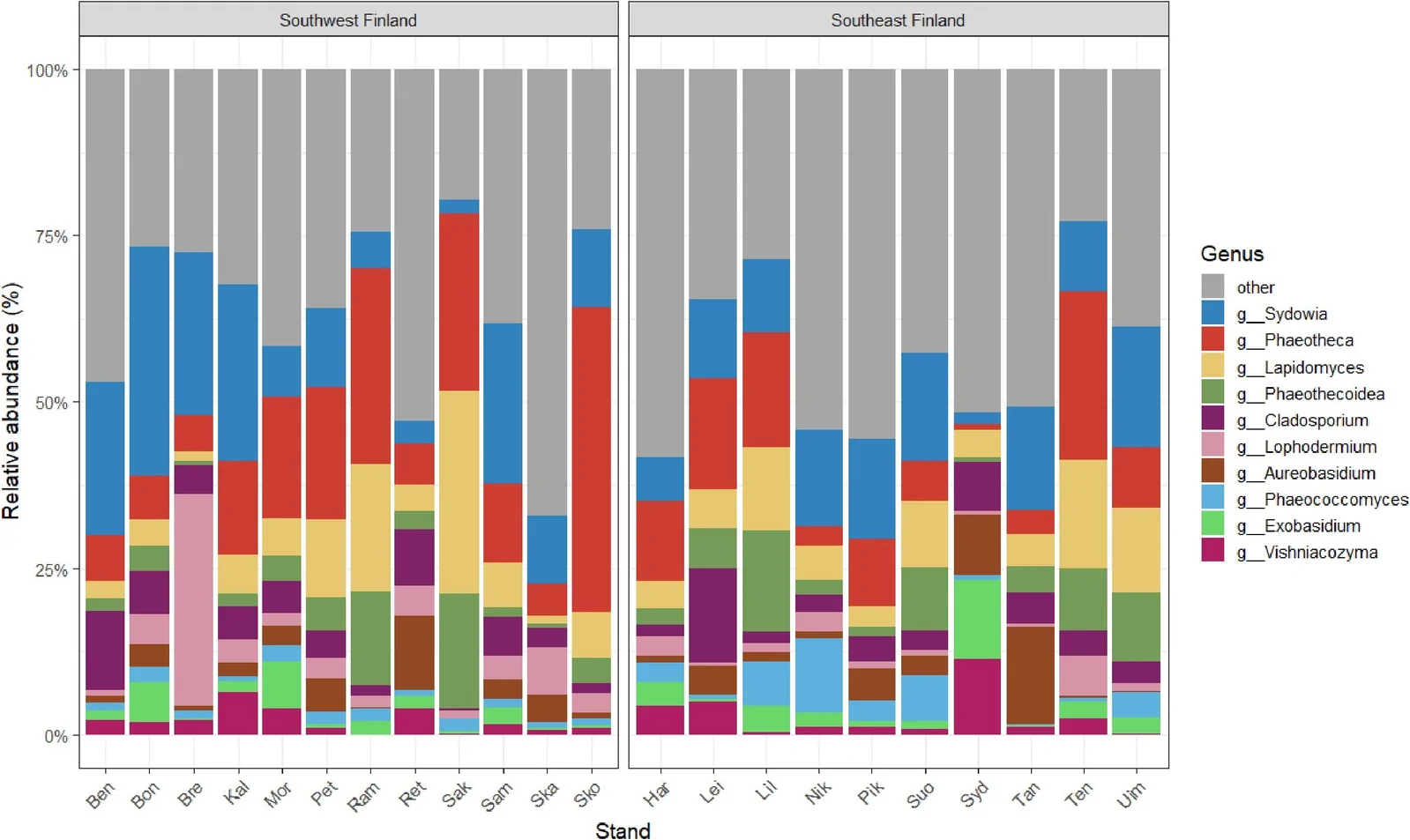
Relative abundance of the 10 most abundant fungal genera across the entire dataset in Southwest Finland (left panel) and Southeast Finland (right panel). Bars represent individual stands, and colors denote taxonomic genera. “Other” includes all genera outside the overall top 10.

### Functional groups

Functional guilds and trophic modes were assigned using FUNGuild, retaining only “Probable” and “Highly probable” assignments while all others were classified as unassigned. This resulted in 1238 OTUs with functional assignments out of a total of 2059 OTUs.

Across both regions, fungal communities were dominated by pathotroph–saprotrophs, followed by saprotrophs and pathotrophs. Stand-level patterns showed moderate variation in trophic composition (Fig. 3), although overall patterns remained broadly consistent across stands.

**Figure 3:**
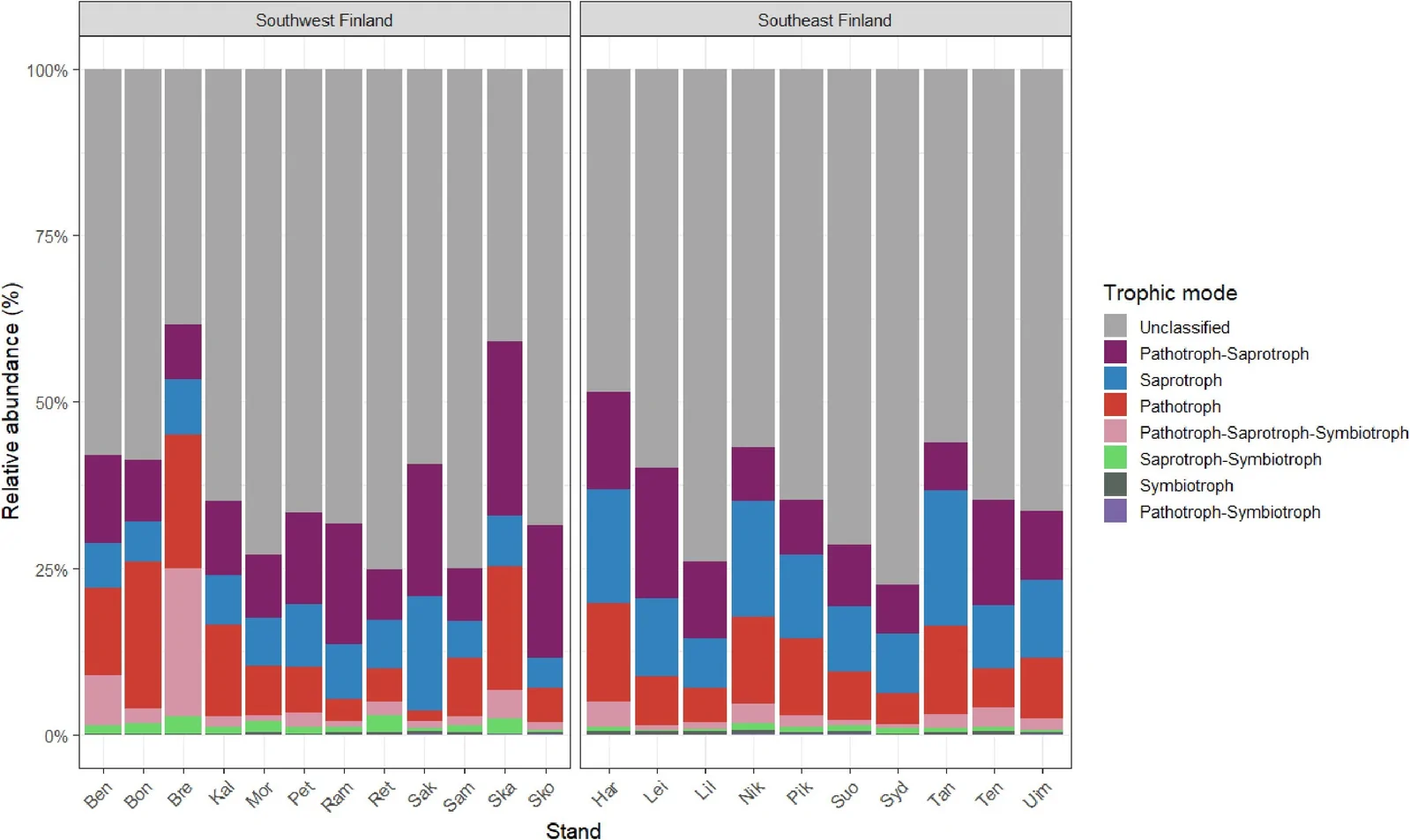
Relative abundance (%) of fungal trophic modes across seedling stands in Southwest Finland (left panel) and Southeast Finland (right panel). Trophic modes were assigned with FUNGuild. Bars represent individual stands, and colors denote different trophic strategies and their combinations. Unclassified taxa are shown in gray.

In Southeast Finland, saprotrophs and pathotroph–saprotrophs represented the largest trophic groups in both natural and planted seedlings, with relatively similar proportions between origins. In contrast, Southwest Finland showed higher relative abundances of pathotrophs and lower proportions of saprotrophs, particularly in planted seedlings (Fig. 4).

**Figure 4:**
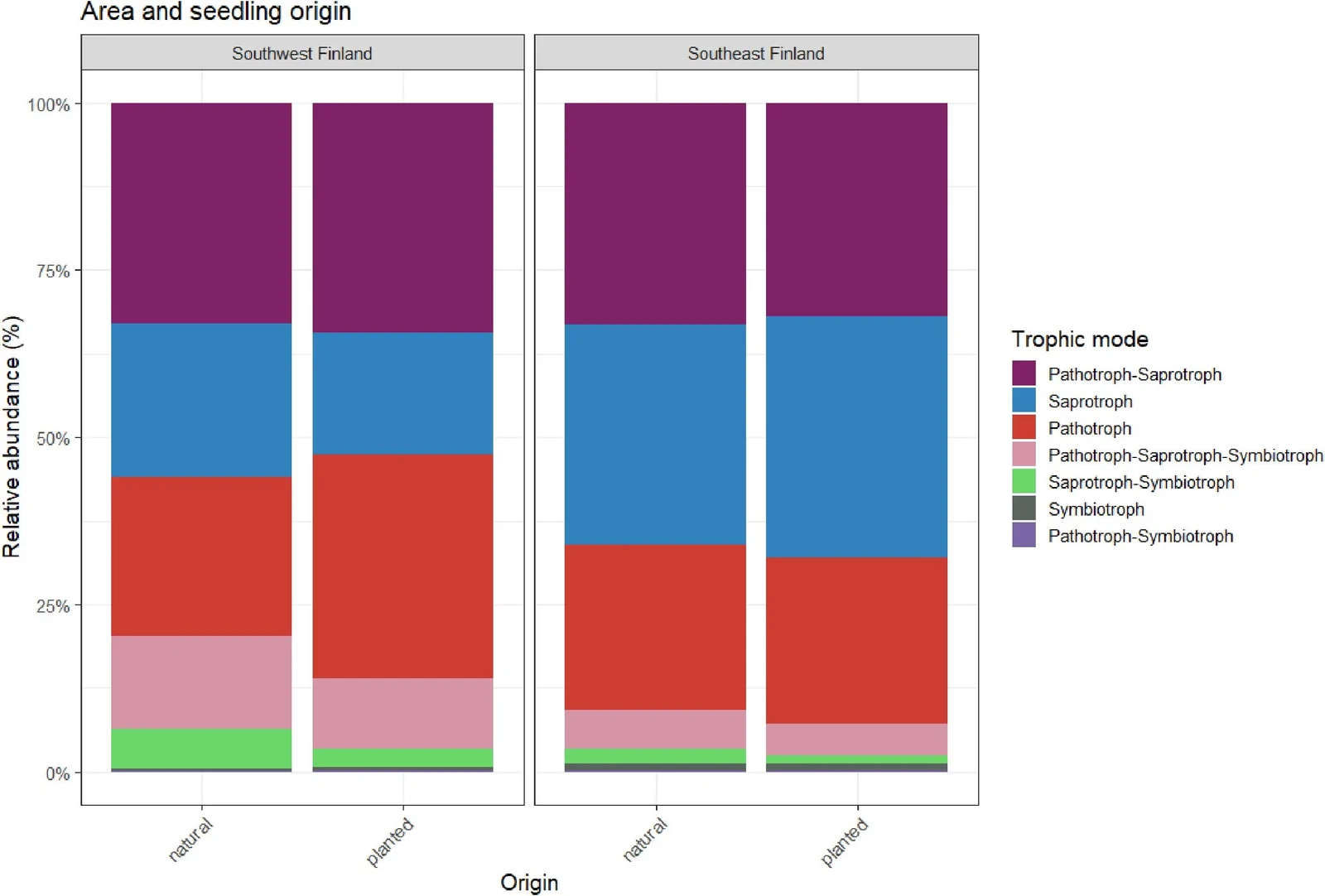
Relative abundance (%) of fungal trophic modes across regions (Southwest and Southeast Finland) and seedling origin (naturally regenerated vs. planted). Only OTUs assigned to at least one trophic mode are shown. Bars show mean relative abundances within each category, and colors represent trophic modes. Based on 1238 OTUs with functional assignments.

FUNGuild assignments revealed a high prevalence of pathogen-associated OTUs in asymptomatic needles (Table 2). In total, 491 OTUs were classified as containing a pathogenic function. Of these, 29% were exclusively assigned to the pathogen guild, while 71% were assigned to multiple ecological guilds (hereafter referred to as multifunctional taxa). Endophyte- and epiphyte-associated OTUs were almost entirely multifunctional (≥95%), whereas saprotroph–associated OTUs showed a more balanced distribution between exclusive and multifunctional assignments. Overall, multifunctional assignments were common across all guilds, highlighting substantial overlap among ecological roles inferred from the literature.

**Table 2:** Functional guild assignments of fungal OTUs inferred using FUNGuild.

Guild	Total OTUs	Single-guild OTUs	Multi-guild OTUs	% multi-guild
**Pathogen**	491	142	349	71.1%
**Saprotroph**	946	450	496	52.4%
**Endophyte**	191	5	186	97.4%
**Epiphyte**	68	3	65	95.6%

OTUs were classified as single-guild or multi-guild based on the number of assigned ecological roles. Percentages indicate the proportion of multi-guild OTUs within each guild.

To illustrate how stand tree species composition relates to functional structure of the foliar mycobiome, we examined correlations between tree species proportion and the abundance of fungal trophic groups. OTUs with multiple trophic assignments showed highly variable and inconsistent associations with tree species composition (Supplementary Figs S2–S3), and groups were not further interpreted due to their ambiguous ecological roles. Main figures therefore focus on single–assignment trophic groups (pathotroph, saprotroph, and symbiotroph).

Pathotroph and saprotroph abundance decreased weakly with increasing birch proportion (ρ = −0.21, *P* = 0.004 and ρ = −0.19, *P* = 0.011, respectively), whereas symbiotroph abundance showed no significant association (ρ = 0.10, *P* = 0.17; Fig. 5a). Conversely, pathotroph and saprotroph abundance showed weak positive associations with increasing spruce proportion (ρ = 0.20, *P* = 0.007 and ρ = 0.18, *P* = 0.013, respectively), while symbiotroph abundance remained unrelated to spruce proportion (ρ = 0.01, *P* = 0.84; Fig. 5b). Thus, significant associations with tree-species composition were observed only for pathotrophs and saprotrophs, whereas symbiotroph abundance showed no significant relationship with either birch or spruce proportion. Despite statistical significance, all observed correlations were weak.

**Figure 5:**
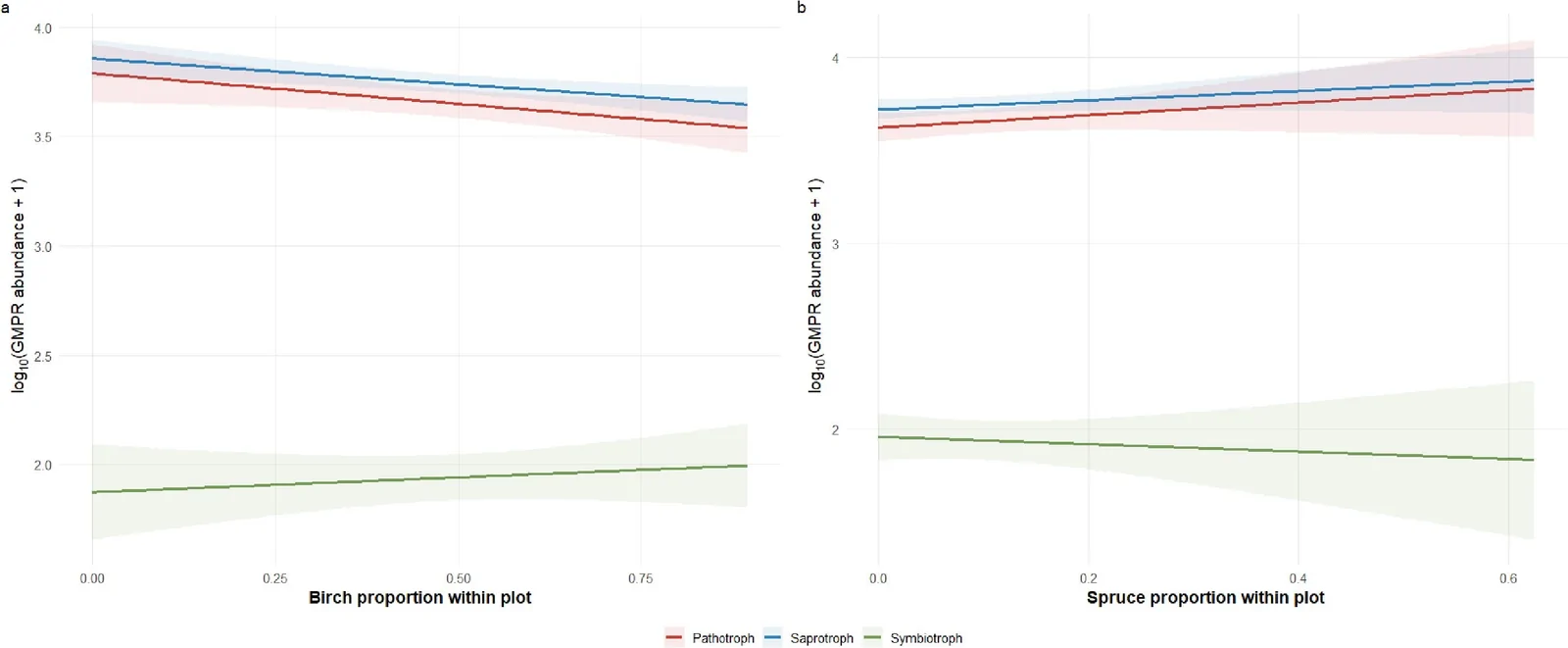
Associations between the proportion of birch (a) and spruce (b) within plots and GMPR-normalized abundances of fungal OTUs with single trophic assignments (pathotroph, saprotroph, and symbiotroph). Lines represent fitted trends with 95% confidence intervals. OTUs assigned to multiple trophic categories are shown in Supplementary Figs S2–S3.

### Alpha diversity

The mixed-effects model explained 15.9% of the marginal variance in Shannon diversity. Origin and geographic area accounted for the largest shares (*R*² = 0.05 and 0.04, respectively), followed by birch proportion and needle C:N ratio (both *R*² = 0.03), and spruce proportion (*R*² = 0.02) (Fig. 6a). Shannon diversity was lower in planted seedlings compared to naturally regenerated ones (*P* = 0.004) and lower in the Southwestern region compared to Southeast (*P* = 0.022). Diversity increased with higher needle C:N ratios (*P* = 0.028) and decreased as birch (*P* = 0.026) and to lesser extent spruce (*P* = 0.050) dominance increased (Fig. 7).

**Figure 6:**
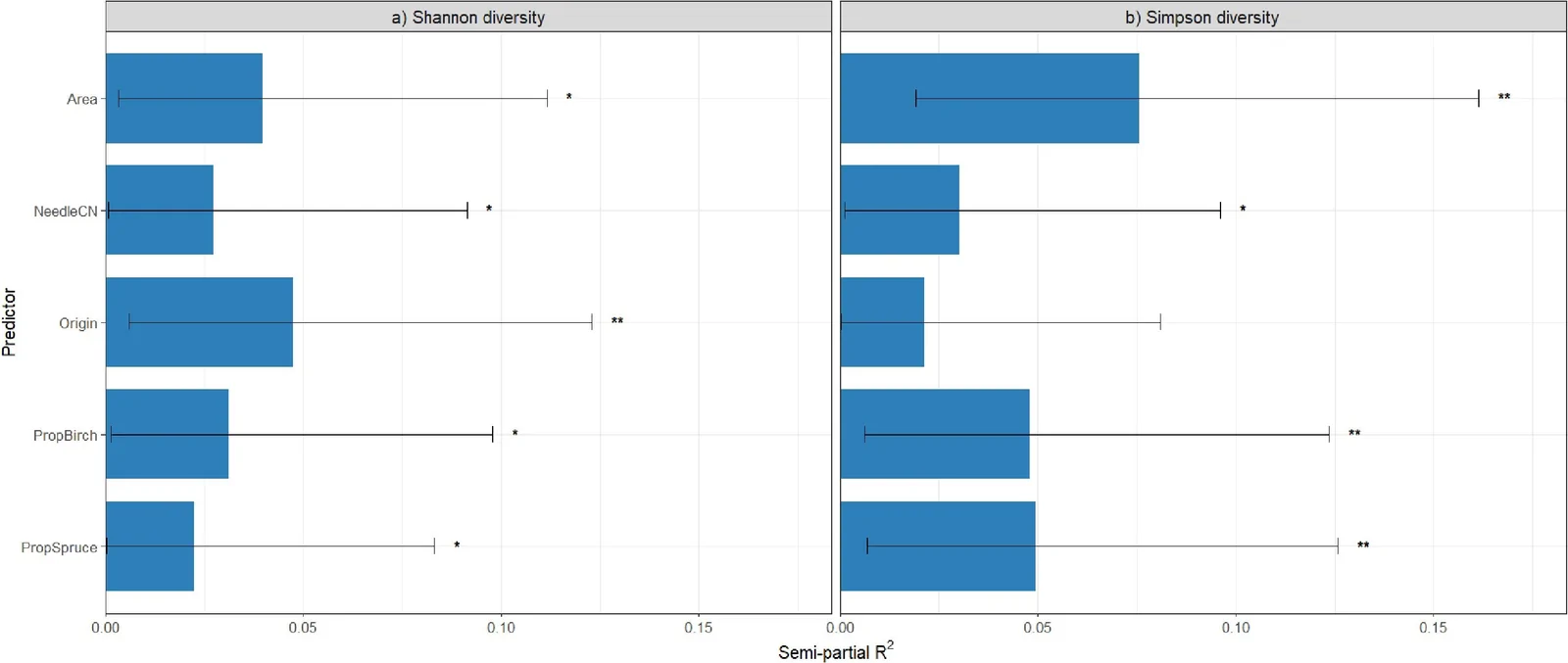
Semi-partial *R*² contributions of predictors in the linear mixed models (LMMs) for (a) Shannon diversity and (b) Simpson diversity. Bars represent the proportion of variance uniquely attributable to each predictor (Area, Needle C:N, Origin, Proportion birch, and Proportion spruce), with horizontal lines indicating 95% confidence intervals. Stars denote significance levels from the LMMs (*P* ≤ 0.05; **P ≤ 0.01; ***P ≤ 0.001).

**Figure 7:**
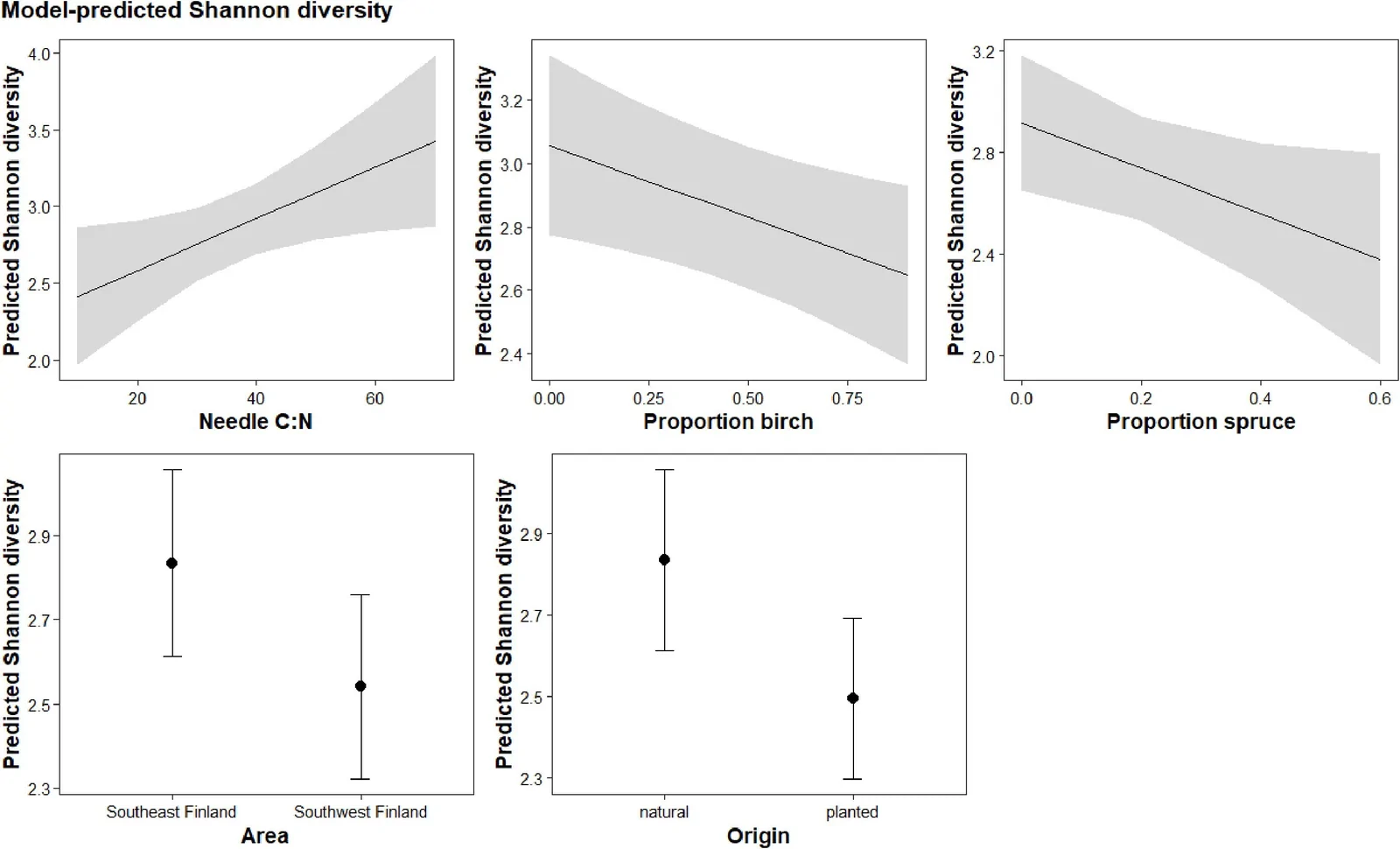
Model predicted Shannon diversity from the LMM. Predicted Shannon diversity from the LMM showing the effects of Needle C:N, Proportion birch, Proportion spruce, Area, and Origin. Lines represent predicted means with 95% confidence intervals for continuous predictors (top panels), and points with error bars indicate predicted group means ±95% CI for categorical predictors (bottom panels). Predictions are shown with Stand included as a random intercept.

Planted seedlings tended to have slightly higher needle nitrogen concentrations and lower C:N ratios than naturally regenerated seedlings, although these differences were not statistically significant. In mixed models, needle C:N ratio was positively associated with Shannon diversity, but its interaction with seedling origin was not significant.

For Simpson diversity, the mixed-effects model explained 17.7% of marginal variance. Geographic area accounted for the largest share (*R*² = 0.08), followed by spruce and birch proportions (both *R*² = 0.05), while needle C:N ratio (*R*² = 0.03) and seedling origin (*R*² = 0.02) contributed less (Fig. 6b). Simpson diversity was lower in the Southwestern region compared to Southeast (*P* = 0.001) and decreased with increasing spruce (*P* = 0.0029) and birch proportions (*P* = 0.0042). Needle C:N ratio showed a weak positive association (*P* = 0.019), whereas seedling origin had a marginal effect (*P* = 0.051), with planted seedlings tending to have lower diversity (Fig. 8)

**Figure 8:**
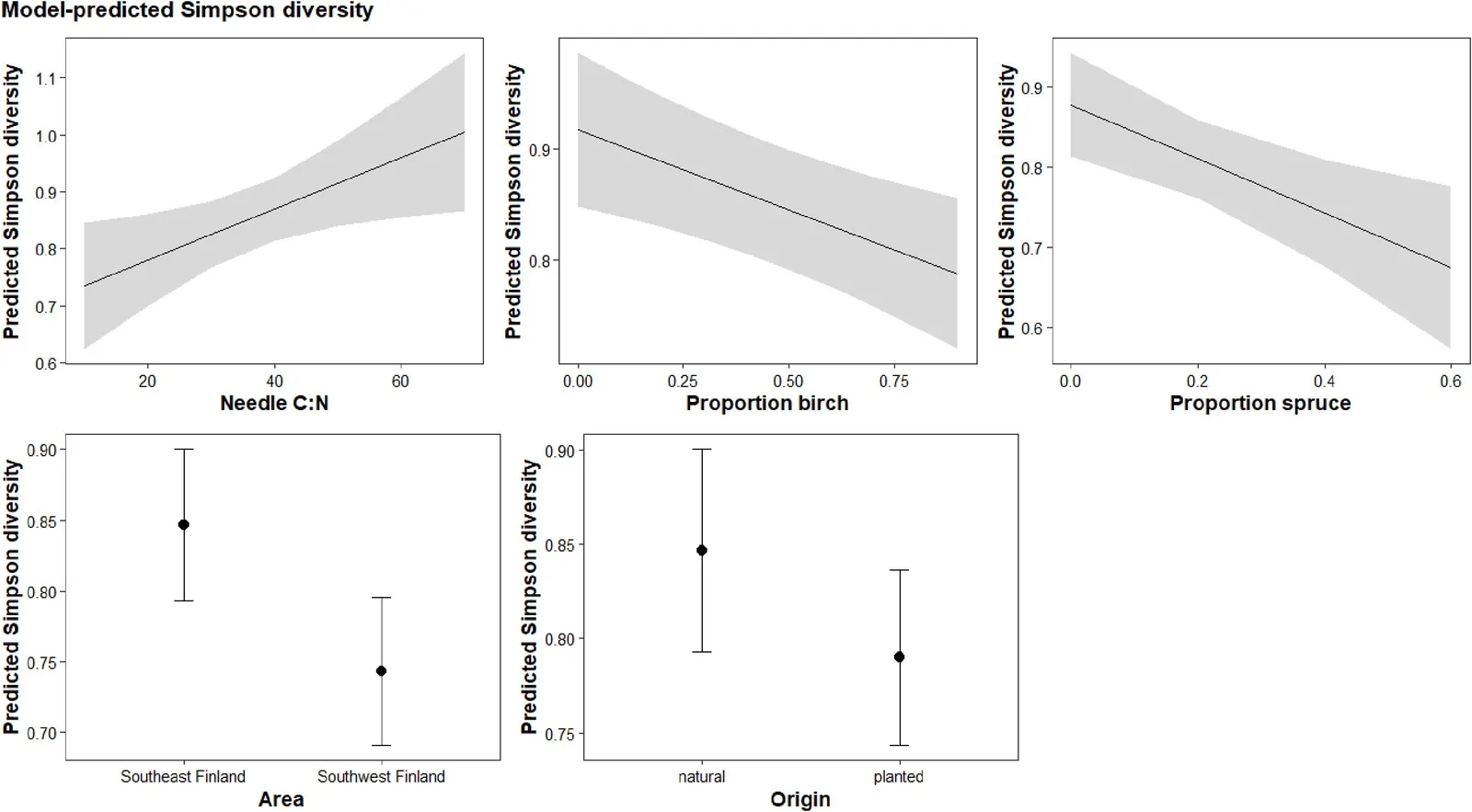
Model predicted Simpson diversity from the LMM. Predicted Simpson diversity from the LMM showing the effects of Needle C:N, Proportion birch, Proportion spruce, Area, and Origin. Lines represent predicted means with 95% confidence intervals for continuous predictors (top panels), and points with error bars indicate predicted group means ±95% CI for categorical predictors (bottom panels). Predictions are shown with Stand included as a random intercept.

Semi-partial *R*² values for all candidate predictors and full model outputs are provided in Supplementary Tables S2–S5. Alpha diversity patterns were consistent between GMPR-normalized and rarefied datasets (Supplementary Tables S10–S11), indicating that sequencing depth did not bias the observed results.

### Beta diversity

NMDS based on Bray–Curtis’s dissimilarities showed moderate fit (0.192 stress) with a clear positive linear relationship between the observed dissimilarity and the ordination distances (for linear fit: *r*^2^ = 0.887; Supplementary Fig. S7). NMDS ordination showed partially overlapping fungal community structures across two geographic regions (Fig. 9a). However, their 95% confidence ellipses differed in both position and extent. Naturally regenerated seedlings showed greater dispersion in the ordination compared to planted seedlings (Fig. 9b), whereas samples from mesic and xeric sites formed highly overlapping clouds of points (Fig. 9c).

**Figure 9:**
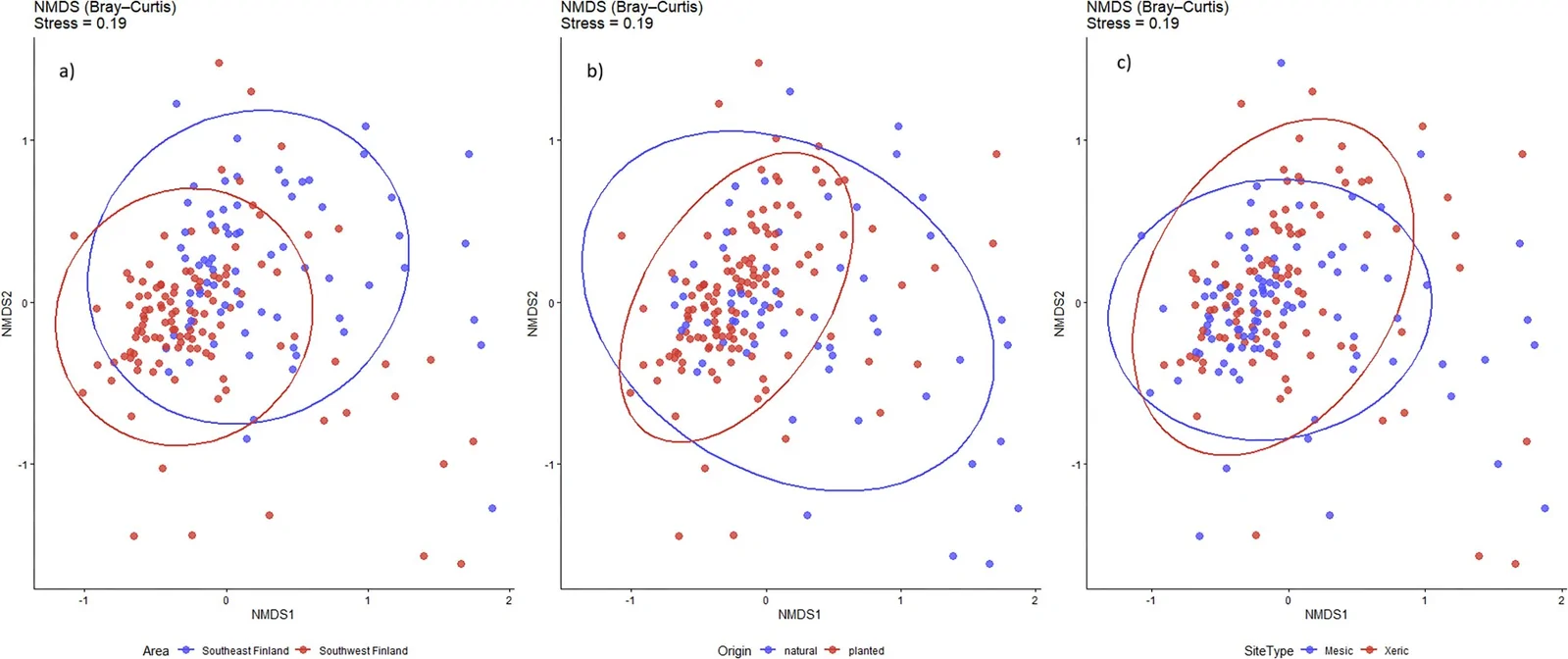
Bray–Curtis NMDS ordinations (stress = 0.19) visualizing fungal community composition colored by (a) Area, (b) Origin, and (c) Site type. Points represent individual samples, and ellipses indicate 95% confidence ellipses around group centroids.

Beta dispersion indicated significant heterogeneity in multivariate variance between naturally regenerated and planted seedlings (*P* = 0.0017). Because of this heterogeneity, the apparent separation between natural and planted seedlings in the ordination cannot reliably be interpreted as a true host–associated shift in community structure. Therefore, origin was excluded from PERMANOVA models. Dispersion did not differ significantly for area or site type.

In a between-stand model, geographic area was the strongest predictor of community composition (*R*² = 0.047, *P* = 0.0001), followed by tree height (*R*² = 0.0260, *P* = 0.0001), birch proportion (*R*² = 0.0153, *P* = 0.0011), spruce proportion (*R*² = 0.0129, *P* = 0.0038), and regeneration year (*R*² = 0.0118, *P* = 0.0071) (Fig. 10a). When variation within stands was examined using restricted permutations (within-stand model), tree height was the dominant predictor (*R*² = 0.031, *P* = 0.0001), while birch proportion showed a smaller but significant effect (*R*² = 0.017, *P* = 0.02) (Fig. 10b). Spruce proportion and regeneration year showed comparable effect size (*R*^2^ = 0.017, *P* = 0.72; *R*^2^ = 0.014, *P* = 0.72), but neither of them were statistically significant when permutations were constrained. Full model outputs for both models are provided in Supplementary Tables S7 and S9.

**Figure 10:**
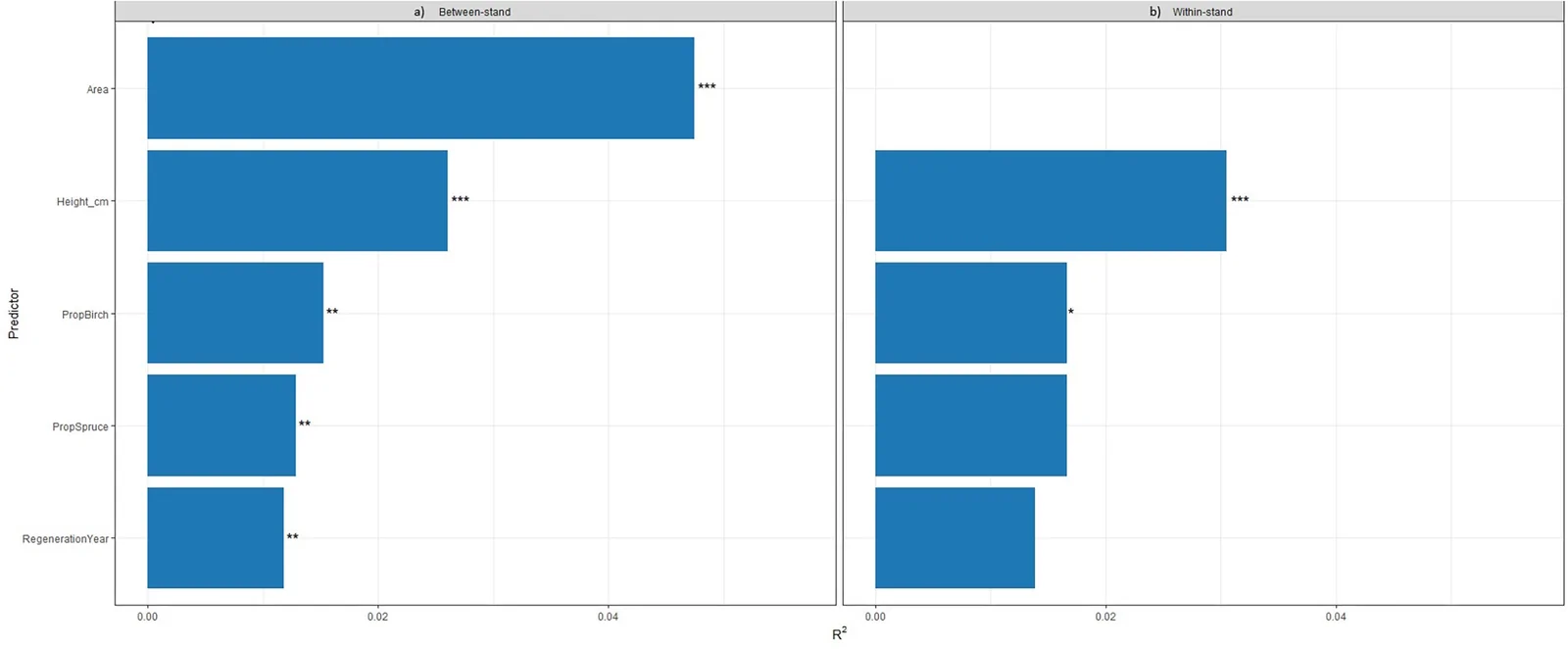
Variance explained (*R*²) by predictors of fungal community composition in the between-stand PERMANOVA model (left) and the within-stand PERMANOVA model with restricted permutations (right). Bars show marginal *R*² values for each predictor. Stars indicate significance levels from marginal PERMANOVA tests (*P* ≤ 0.05; *P* ≤ 0.01; *P* ≤ 0.001).

PERMANOVA results were consistent when analyses were repeated using rarefied data (Supplementary Tables S12–S13 and Fig. S11), indicating that the observed patterns in community composition were robust to variation in sequencing depth.

### Differential abundance analysis

Differential–abundance analysis using ANCOM–BC2 identified several genera that showed significant (*q* < 0.05) shifts between natural and planted seedlings in the unfiltered output (Supplementary Fig. S8), but none of these taxa passed the ANCOM-BC2 pseudo-sensitivity analysis. Consequently, no robust differentially abundant taxa were confirmed.

In contrast, 103 differentially abundant OTUs representing 55 genera showed significant shifts between geographic regions (Supplementary Fig. S9); and 11 OTUs from 9 genera remained significant after the pseudo–count sensitivity filter. Genera *Kabatiella, Rachicladosporium, Friedmanniomyces, Ramularia, Sporocadus, Venturia*, and *Pucciniastrum* were more abundant in Southeast Finland, while only one genus, *Symmetrospora*, showed higher abundance in Southwest Finland (Fig. 11).

**Figure 11:**
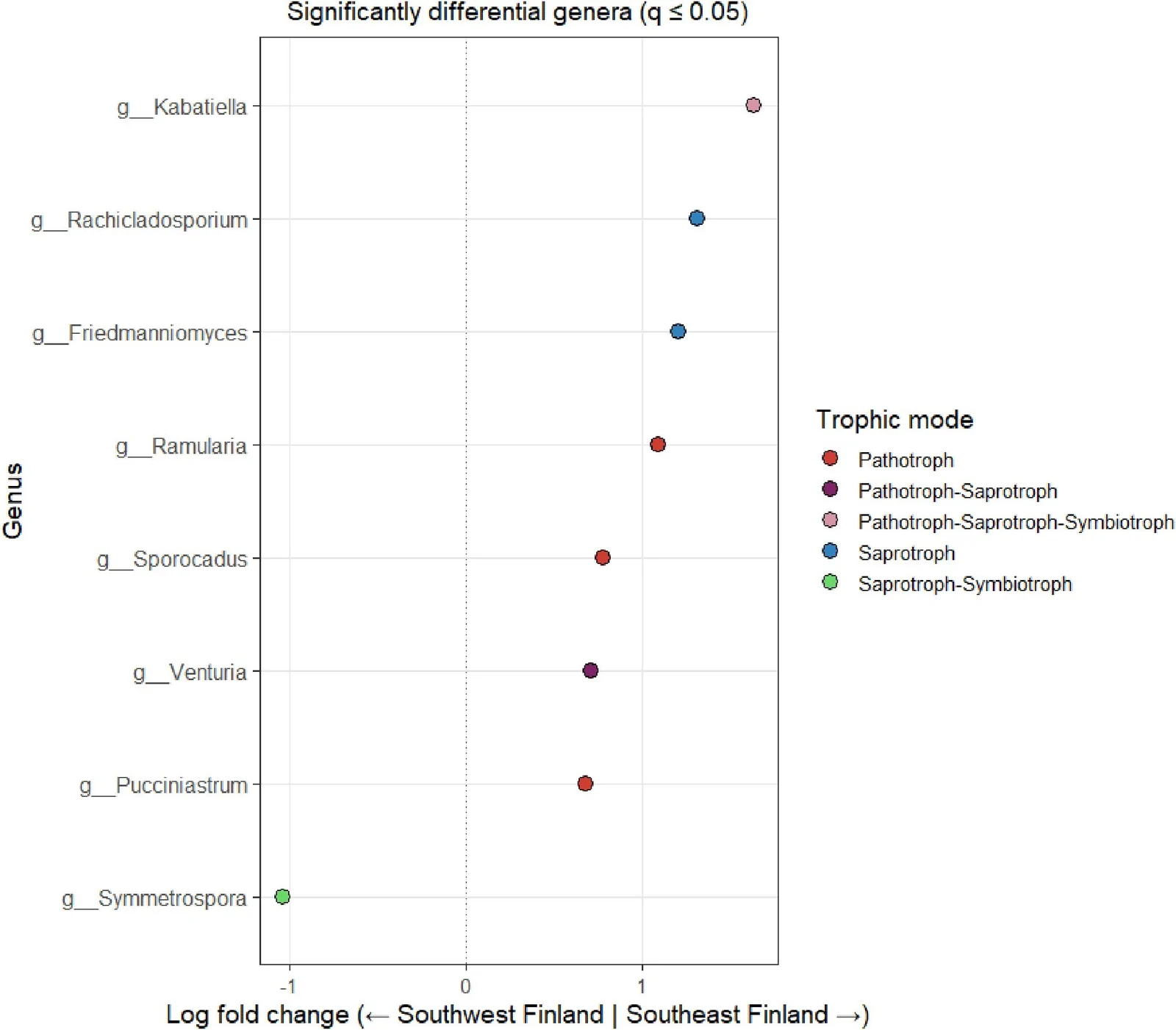
Genus-level differential abundance between geographic regions identified using ANCOM-BC2. Only genera passing both the *q* < 0.05 significance threshold and the pseudo-count sensitivity filter are shown. Negative log-fold changes indicate higher abundance in Southwest Finland, whereas positive values indicate higher abundance in Southeast Finland. Colors indicate trophic guild assignments.

## Discussion

Our results show that early foliar mycobiomes of Scots pine seedlings are shaped by a combination of regional biogeography, seedling origin, stand composition and needle nitrogen status. Across all sites, fungal communities were dominated by a small number of abundant taxa accompanied by large pool of rare OTUs.

Despite sampling only seedlings without visible disease symptoms, many OTUs were classified as having pathogenic functions. This finding is consistent with previous studies showing that fungi with recognized pathogenic potential may occur in asymptomatic plant tissues (Delaye et al. 2013, Indermaur and Westrick 2026). However, because needles were not surface sterilized prior to DNA extraction, our approach does not allow us to distinguish fungi colonizing internal tissues from those occurring on needle surface. These interpretations should also be treated with caution given the high proportion of unclassified taxa and the known limitations of FUNGuild assignments, particularly for taxa with context-dependent ecological roles.

### Geographic region as a primary driver of community structure

Consistent with our first hypothesis, geographic region shaped both fungal diversity and community composition. It explained the largest share of variation in Simpson diversity and accounted for the greatest proportion of explained variation in community composition in the between-stand PERMANOVA model. Seedlings in southwestern Finland had lower fungal diversity than those in southeastern Finland, and community composition differed significantly between the two regions. However, the partial overlap observed in the NMDS ordination indicates that the regional communities were not completely distinct. The study regions differ substantially in landscape setting, with the Southwestern archipelago representing a more maritime environment and Southeastern Finland a predominantly inland landscape. Although environmental variables were not directly measured in this study, the observed regional differences are consistent with earlier studies showing latitudinal and longitudinal variation in Scots pine needle mycobiomes, likely driven by differences in climate (temperature and precipitation) and regional species pools (Terhonen et al. 2011, Millberg et al. 2015).

Regional differences were most strongly reflected in the relative abundances of dominant taxa, while functional guild composition was broadly similar between regions. Differential abundance analysis identified only a small number of genera that were robustly associated with specific regions. Together these results suggest that regional differences were not driven by a few indicator taxa but rather by broader shifts across many taxa, consistent with regional species turnover and changes in community dominance patterns.

### Stand composition modifies dominance patterns

Our findings support the second hypothesis that surrounding tree species composition contributes to the assembly of Scots pine foliar fungal communities, while also showing that the strength and nature of these effects depend on spatial scale. Community composition varied substantially among stands within both regions, with some stands characterized by strong dominance of a single taxon and others showing more even distributions among multiple taxa. This within-region heterogeneity, together with the contrasting results between the broad-scale (between-stand) and fine-scale (within-stand) PERMANOVA models, highlight the scale dependency of ecological drivers shaping the foliar fungal community composition. At broader spatial scales, community composition was primarily structured by geographic region together with stand-level characteristics such as tree species composition and stand age. However, when variation among stands was accounted for, local host-related and neighborhood-level effects became dominant.

Seedling height emerged as the strongest predictor of community composition within stands, suggesting that factors associated with variation in seedling performance, growth history, and local environmental conditions may contribute to the assembly of foliar fungal communities. Similarly, fungal endophyte richness was found to reflect the current growth rate of juvenile Norway spruce clones (Rajala et al. 2014). The consistent association between birch proportion and fungal communities across both spatial scales suggests that birch may influence fungal communities not only through overall stand composition, but also through local environmental conditions and fungal dispersal opportunities. However, the observational nature of this study does not allow these mechanisms to be tested directly. In contrast, spruce proportion and regeneration year were only influential at the between-stand level, indicating that their effects mainly reflect broader differences among stands rather than fine-scale variation within them.

While the PERMANOVA analyses demonstrated scale-dependent effects on community composition, alpha diversity analyses revealed a simpler pattern. Increasing proportions of birch and spruce were associated with reduced fungal diversity, suggesting that neighboring tree species can influence which fungal taxa are able to colonize and persist in pine needles.

Despite these clear effects on taxonomic diversity and community composition, corresponding changes in functional guild structure were less pronounced. Although some trophic groups showed trends along birch and spruce gradients, these relationships were generally weak and inconsistent, and many OTUs were assigned to multiple ecological guilds. This suggests substantial functional redundancy among taxa, whereby changes in species identity do not necessarily translate into major shifts in the overall functional structure of the foliar mycobiome.

These findings are consistent with previous studies showing that both tree species richness and species identity shape foliar microbial communities through mechanisms such as microclimatic modification, host dilution, and altered dispersal dynamics (Hantsch et al. 2014, Field et al. 2020). In our study, higher birch proportion was associated with reduced abundance of both saprotrophic and pathotrophic fungi. This may reflect a neighbor-mediated dilution effect, where non-host broadleaf trees reduce the availability or transmission of pine-associated fungi. In contrast, spruce showed the opposite pattern, possibly reflecting greater overlap in fungal communities among conifer hosts, which may facilitate the persistence of pine-associated fungi in mixed conifer stands (Wang et al. 2024). Similar processes have been observed in forest ecosystems, where increasing tree diversity reduces pathogen damage while maintaining, or even increasing, overall fungal diversity at the community level (Rutten et al. 2021).

### Seedling origin affects diversity and community variability

Contrary to our third hypothesis, foliar fungal communities differed between naturally regenerated and planted seedlings, although this effect was limited to Shannon diversity. Planted seedlings exhibited lower Shannon diversity compared to naturally regenerated seedlings, suggesting reduced representation of rare taxa, whereas Simpson diversity showed only a marginal association with seedling origin. Differences in community composition between the two origins could not be evaluated reliably because naturally regenerated seedlings exhibited significantly greater multivariate dispersion than planted seedlings. This greater among-sample variability indicates that fungal communities were more heterogenous among naturally regenerated seedlings, whereas communities in planted seedlings were comparatively more similar. Consequently, it remains unclear whether seedling origin influences overall community composition, although the diversity results suggest that its effects may be most pronounced among less abundant members of the foliar fungal community.

Previous work has shown that foliar fungal communities vary over the growing season, with needle age strongly influencing community composition (Oono et al. 2015, Agan et al. 2021, Larsson et al. 2023, López-Fernández 2023). This suggests that similar exposure time to local inoculum could lead toward similar foliar fungal communities, regardless of origin. However, because community composition could not be tested independently of differences in multivariate dispersion, this interpretation should be viewed with caution.

Nevertheless, the lower Shannon diversity observed in planted seedlings indicates that seedling origin still influences community assembly. Although planted seedlings tended to have slightly higher needle nitrogen concentrations and lower C:N ratios than naturally regenerated seedlings, these differences were not statistically significant. Furthermore, the non-significant interaction between seedling origin and needle C:N ratio suggests that reduced diversity in planted seedlings is not explained by needle nitrogen status alone. The persistence of diversity differences despite broadly similar needle chemistry indicates that other host-associated factors may contribute to community assembly.

One possible explanation is that naturally regenerated seedlings differ in their belowground microbial communities. Root-associated microbes can influence aboveground microbial communities through plant-mediated effects on nutrient allocation, defense responses, and tissue chemistry (Hassani et al. 2018, Tedersoo and Bahram 2019, Marčiulynienė et al. 2022). Because naturally regenerated seedlings develop under local soil conditions, they may harbor root microbiomes distinct from those of nursery-grown seedlings. Nursery practices such as fertilization and fungicide treatments can alter fungal communities and shift ectomycorrhizal composition (Smaill and Walbert 2013), and these effects can persist after outplanting for multiple years (Franco et al. 2014). Such shifts in mycorrhizal communities can have lasting ecological consequences through their effects on plant nutrient uptake and performance (Smaill and Walbert 2013, Franco et al. 2014). Recent studies have further highlighted links between belowground symbionts and foliar fungal communities in trees (Köhler et al. 2025a; Ariyan et al. 2026), providing a potential mechanism for the patterns observed here.

Together, these results suggest that current-year needle mycobiomes are shaped not only by recent colonization from the local environment but also by the developmental history of the host. While the effects of seedling origin on overall community composition remain uncertain, the consistently lower Shannon diversity observed in planted seedlings indicates that origin may influence the persistence and establishment of less abundant fungal taxa.

### Needle nitrogen status shapes diversity

Our fourth hypothesis was partly fulfilled, as needle C:N ratio was positively associated with fungal alpha diversity but not with beta diversity. Seedlings with higher C:N ratios, which in our dataset primarily reflected lower needle nitrogen concentrations, tended to support more diverse fungal communities, whereas community composition remained largely unchanged.

The mechanisms underlying this relationship remain unclear. Because the observed variation reflects naturally occurring differences among largely healthy seedlings rather than experimentally manipulated nutrient conditions, our results should not be interpreted as evidence of nitrogen limitation. Nevertheless, variation in needle nitrogen status may alter resource availability within needles, potentially influencing the competitive balance among fungal taxa and thereby affecting community diversity. For example, resource-limited environments may limit the growth of fast-growing opportunistic fungi, thereby reducing competitive dominance and allowing more species to coexist (Harpole et al. 2016). Previous studies have also shown that leaf functional traits, particularly nitrogen availability, can influence fungal richness, although the direction of these effects can vary depending on host species and environmental context (Tellez et al. 2022, Köhler et al. 2025b)

In this context, our results suggest that variation in needle nitrogen status may contribute to fungal diversity patterns without substantially altering overall community composition. Importantly, the absence of a clear beta-diversity response indicates that variation in C:N ratio was associated primarily with diversity within a shared species pool rather than with compositional turnover among sites.

### Conclusion

Overall, our results show that the Scots pine seedling needle mycobiome is structured by multiple ecological drivers operating at different spatial scales. At the broadest scale, geographic region primarily structured the dominant components of the fungal community, whereas seedling origin was associated with variation in less abundant taxa. At finer spatial scales, fungal diversity and community composition were associated with local tree species composition and seedling height, whereas needle nitrogen status was associated only with fungal diversity. In addition, the co-occurrence of taxa assigned to multiple trophic modes highlights the ecological flexibility of many needle-associated fungi. Such flexibility may become particularly relevant under environmental stress (e.g. drought), potentially altering host–microbe interactions, with implications for forest health under changing climate and forest management regimes.

These findings suggest that regeneration method and stand tree species composition are associated with differences in needle fungal communities already during the early stages of seedling establishment. More broadly, our results indicate that different ecological drivers shape different components of the needle mycobiome, highlighting potential links between management-related stand characteristics and fungal community composition long before any visible symptoms of disease emerge.

## Supplementary Material

fiag114_Supplemental_Files

## Data Availability

All data and Supplementary material are available in Zenodo: (https://doi.org/10.5281/zenodo.20589402). The raw data for this study have been deposited in the European Nucleotide Archive (ENA) at EMBL-EBI under accession number PRJEB114804 (https://www.ebi.ac.uk/ena/browser/view/PRJEB114804) and will be publicly released upon publication.
