# Supplementary material for: Regional species turnover and local host and forest stand characteristics jointly structure early foliar mycobiomes of Scots pine seedlings
Source: FEMS Microbiol Ecol. 2026 Oct 5;102(11):fiag114. doi: 10.1093/femsec/fiag114 (PMC13673988; doi:10.1093/femsec/fiag114)
Supplement: Supplementary file 1 [file fiag114_supplemental_files.zip › Supplementary material_REVISED.docx]

Supplementary material

This document contains supplementary tables and figures supporting the main analyses. The content is organized by analysis type:

Contents

[**Section 1: Sampling effort and stand characteristics**	2](#_Toc238484930)

[**Section 2: Community composition**	4](#_Toc238484931)

[**Section 3: Functional analyses**	5](#_Toc238484932)

[**Section 4: Alpha diversity**	7](#_Toc238484933)

[**Section 5: Beta diversity**	15](#_Toc238484934)

[**Section 6: Differential abundance**	17](#_Toc238484935)

[**Section 7: Results with rarefied data**	19](#_Toc238484936)

## **Section 1: Sampling effort and stand characteristics**

*Table S1. Overview of sampling design, regeneration year and stand composition across study sites in southern Finland. For each stand, the table reports the geographic area, stand identifier, year of regeneration, number of sampling plots (n), total number of measured pine seedlings, and number of collected needle samples. Proportion of pine seedlings sampled indicates the fraction of measured pine seedlings from which needle samples were obtained. Proportion of pine, spruce, and birch represent stand-level species composition, calculated as the relative proportions of each tree species based on all recorded individuals across sampling plots within a stand. Mean pine height (cm) denotes the average height of all measured pine seedlings across all sampling plots within each stand*

| Area | Stand | Year of regeneration | Plot  (n) | Pine seedlings measured (n) | Needle samples (n) | Proportion of pine seedlings sampled | Proportion of  Scots pine | Proportion of Norway spruce | Proportion of silver/downy birch | Mean height of pine seedlings (cm) |
| --- | --- | --- | --- | --- | --- | --- | --- | --- | --- | --- |
| Kanta-Häme | Suo | 2023 | **3** | **15** | **6** | 0.4 | 0.56 | 0.38 | 0.07 | 44.41 |
| Southeast  Finland | Har | 2021 | **2** | **36** | **6** | 0.17 | 0.69 | 0.05 | 0.26 | 36.08 |
| Southeast  Finland | Lei | 2022 | **3** | **19** | **9** | 0.47 | 0.59 | 0.03 | 0.38 | 46.30 |
| Southeast  Finland | Lil | 2021 | **3** | **29** | **9** | 0.31 | 0.18 | 0 | 0.82 | 90.19 |
| Southeast  Finland | Nik | 2021 | **3** | **18** | **9** | 0.5 | 0.27 | 0.13 | 0.60 | 51.74 |
| Southeast  Finland | Pik | 2022 | **2** | **14** | **6** | 0.43 | 0.30 | 0.41 | 0.29 | 39.18 |
| Southeast  Finland | Syd | 2020 | **2** | **26** | **6** | 0.23 | 0.31 | 0.28 | 0.41 | 68.47 |
| Southeast  Finland | Tan | 2021 | **1** | **5** | **3** | 0.6 | 0.23 | 0.5 | 0.27 | 70.34 |
| Southeast  Finland | Ten | 2023 | **2** | **13** | **6** | 0.46 | 0.86 | 0.14 | 0 | 39.67 |
| Southeast  Finland | Uim | 2018 | **3** | **39** | **9** | 0.23 | 0.32 | 0.25 | 0.43 | 74.43 |
| Southeast  Finland | Vas | 2019 | **3** | **35** | **9** | 0.26 | 0.49 | 0.10 | 0.41 | 67.84 |
| Southwest  Finland | Ben | 2022 | **3** | **13** | **8** | 0.62 | 0.58 | 0.21 | 0.21 | 37.85 |
| Southwest  Finland | Bon | 2021 | **4** | **39** | **13** | 0.33 | 0.22 | 0.01 | 0.77 | 54.56 |
| Southwest  Finland | Bre | 2020 | **3** | **20** | **9** | 0.45 | 0.38 | 0.18 | 0.44 | 71.57 |
| Southwest  Finland | Kal | 2020 | **5** | **91** | **16** | 0.18 | 0.48 | 0.01 | 0.5 | 59.06 |
| Southwest  Finland | Mor | 2020 | **4** | **28** | **12** | 0.43 | 0.13 | 0.02 | 0.85 | 57.55 |
| Southwest  Finland | Pet | 2021 | **4** | **41** | **12** | 0.29 | 0.37 | 0.08 | 0.55 | 38.31 |
| Southwest  Finland | Ram | 2019 | **3** | **31** | **9** | 0.29 | 0.29 | 0.03 | 0.68 | 114.54 |
| Southwest  Finland | Ret | 2023 | **1** | **12** | **3** | 0.25 | 0.28 | 0.0 | 0.72 | 41.79 |
| Southwest  Finland | Sak | 2020 | **1** | **9** | **3** | 0.33 | 0.82 | 0.0 | 0.18 | 92.00 |
| Southwest  Finland | Sam | 2020 | **4** | **27** | **12** | 0.44 | 0.45 | 0.25 | 0.3 | 62.96 |
| Southwest  Finland | Sko | 2019 | **4** | **29** | **12** | 0.41 | 0.23 | 0.01 | 0.76 | 101.20 |
| Southwest  Finland | Ska | 2021 | **2** | **7** | **6** | 0.86 | 0.23 | 0.1 | 0.67 | 49.90 |

## **Section 2: Community composition**

*Figure S1. Relative abundance of the 10 most abundant fungal families across the entire dataset in Southwest Finland (left panel) and Southeast Finland (right panel). Bars represent individual stands, and colors denote taxonomic families. “Other” includes all families outside the overall top 10.*

## **Section 3: Functional analyses**

*Figure S2. Relationships between the abundance of OTUs assigned to multiple trophic guilds and birch proportion within plots. Points represent plot-level observations, coloured lines indicate fitted linear trends, and shaded areas represent 95% confidence intervals. Spearman's correlation coefficients (ρ) and associated p-values are shown within each panel.*

Figure S3. Relationships between the abundance of OTUs assigned to multiple trophic guilds and spruce proportion within plots. Points represent plot-level observations, coloured lines indicate fitted linear trends, and shaded areas represent 95% confidence intervals. Spearman's correlation coefficients (ρ) and associated p-values are shown within each panel

## **Section 4: Alpha diversity**

*Table S2. Initial variable screening semi‑partial R² table for Shannon diversity (full model with all candidate predictors). Bootstrapped 95% confidence intervals are shown for completeness. Variables that were retained for the final model are bolded.*

| **Effect** | **Rsq** | **CL upper** | **CL lower** |
| --- | --- | --- | --- |
| **Model** | **0.169** | **0.296** | **0.111** |
| **Area** | **0.037** | **0.108** | **0.003** |
| **Needle C:N** | **0.033** | **0.101** | **0.002** |
| **Origin** | **0.022** | **0.083** | **0.000** |
| **Spruce proportion** | **0.021** | **0.080** | **0.000** |
| **Birch proportion** | **0.019** | **0.077** | **0.000** |
| Height, cm | 0.007 | 0.050 | 0.000 |
| Regeneration year | 0.001 | 0.031 | 0.000 |
| SiteType | 0.000 | 0.028 | 0.000 |

*Table S3. Coefficient table for Shannon LMM.*

| Model Shannon: Shannon ~ Area + Origin + NeedleCN + PropSpruce + PropBirch + (1 \| Stand) | | | | |
| --- | --- | --- | --- | --- |
| Fixed Effect | **Estimate** | **Std. Error** | **t value** | **p-value** |
| (Intercept) | 2.548 | 0.306 | 8.33 | <0.001 *** |
| Area (SW Finland) | -0.294 | 0.119 | -2.48 | 0.022 * |
| Origin (Planted) | -0.340 | 0.117 | -2.92 | 0.004 ** |
| Needle C:N | 0.017 | 0.008 | 2.22 | 0.028 * |
| PropSpruce | -0.894 | 0.450 | -1.99 | 0.050 * |
| PropBirch | -0.455 | 0.199 | -2.29 | 0.026 * |

*Figure S4. Diagnostic plots for the linear mixed-effects model of Shannon diversity. (A) Residuals versus fitted values, (B) normal Q–Q plot of residuals, (C) distribution of residuals, and (D) Q–Q plot of stand-level random effects. Residuals were approximately normally distributed with no strong heteroscedasticity, indicating adequate model fit.*

*Table S4. Initial variable screening semi partial R² table for Simpson diversity*

*(full model with all candidate predictors). Bootstrapped 95% confidence intervals are shown for completeness. Variables that were retained for the final model are bolded.*

| **Effect** | **Rsq** | **CI upper** | **CI lower** |
| --- | --- | --- | --- |
| **Model** | **0.176** | **0.302** | **0.114** |
| **Area** | **0.067** | **0.149** | **0.015** |
| **Spruce, %** | **0.045** | **0.119** | **0.005** |
| **Birch, %** | **0.040** | **0.112** | **0.004** |
| **Needle C:N** | **0.030** | **0.096** | **0.001** |
| **Origin** | **0.013** | **0.064** | **0.000** |
| Height, cm | 0.000 | 0.029 | 0.000 |
| Site type | 0.000 | 0.029 | 0.000 |
| Regeneration year | 0.000 | 0.027 | 0.000 |

*Table S5. Coefficient table for Simpson LMM.*

| Model Simpson: Simpson ~ Area + Origin + NeedleCN + PropSpruce + PropBirch + (1 \| Stand) | | | | |
| --- | --- | --- | --- | --- |
| Fixed Effect | **Estimate** | **Std. Error** | **t value** | **p-value** |
| (Intercept) | 0.791 | 0.076 | 10.46 | < 2e-16 *** |
| Area (SW Finland) | -0.104 | 0.028 | -3.74 | 0.001 ** |
| Origin (Planted) | -0.057 | 0.029 | -1.98 | 0.051 |
| Needle C:N | 0.005 | 0.002 | 2.37 | 0.019 * |
| PropSpruce | -0.339 | 0.111 | -3.05 | 0.003 ** |
| PropBirch | -0.144 | 0.048 | -2.98 | 0.004 ** |

*Figure S5. Diagnostic plots for the linear mixed-effects model of Simpson diversity. (A) Residuals versus fitted values, (B) normal Q–Q plot of residuals, (C) distribution of residuals, and (D) Q–Q plot of stand-level random effects. Residuals were approximately normally distributed with no strong heteroscedasticity, indicating adequate model fit.*

*Figure S6. Variance inflation factors (VIF) for predictors included in the linear mixed-effects models of alpha diversity. Points represent VIF estimates with 95% confidence intervals. All predictors showed low collinearity (VIF < 1.5), indicating no evidence of problematic multicollinearity.*

## **Section 5: Beta diversity**

*Figure S7. Stressplot showing the relationship between observed Bray–Curtis dissimilarities and ordination distances from the NMDS model. The blue points represent pairwise sample comparisons, and the red line shows a linear fit (R² = 0.887). The high non‑metric R² indicates a good ordination fit to the rank order of community dissimilarities.*

*Table S6. R² values from the full Between-Stand PERMANOVA model used to screen predictors for inclusion in the final reduced model.*

| **Effect** | **R^2^** |
| --- | --- |
| **Model** | **0.151** |
| **Area** | **0.045** |
| **Height, cm** | **0.026** |
| **Birch, %** | **0.015** |
| **Spruce, %** | **0.013** |
| **Regeneration year** | **0.012** |
| Site type | 0.007 |
| NeedleCN | 0.005 |

*Table S7. Between-Stand PERMANOVA results for fungal community composition (Bray–Curtis).*

| Between-Stand PERMANOVA model: dist ~ Area + PropBirch + PropSpruce + Height_cm + RegenerationYear; 9999 permutations; by = “margin” | | | | | |
| --- | --- | --- | --- | --- | --- |
| Predictor | **Df** | **Sum Of Squares** | **R²** | **F** | **P Value** |
| Area | 1 | 1.842 | 0.04745 | 9.7485 | 0.0001 *** |
| Propbirch | 1 | 0.593 | 0.01526 | 3.1358 | 0.0011 ** |
| Propspruce | 1 | 0.499 | 0.01286 | 2.6427 | 0.0038 ** |
| Height_cm | 1 | 1.010 | 0.02602 | 5.3456 | 0.0001 *** |
| Regenerationyear | 1 | 0.459 | 0.01183 | 2.4304 | 0.0071 ** |
| Residual | 177 | 33.449 | 0.86155 |  |  |
| Total | 182 | 38.824 | 1.00000 |  |  |

*Table S8. R ² values from the full Within-Stand PERMANOVA model used to screen predictors for inclusion in the final reduced model.*

| **Effect** | **R^2^** |
| --- | --- |
| **Model** | **0.106** |
| **Height, cm** | **0.030** |
| **Birch, %** | **0.016** |
| **Spruce, %** | **0.016** |
| **Regeneration year** | **0.014** |
| Site type | 0.008 |
| NeedleCN | 0.007 |

*Table S9. Within-Stand PERMANOVA results with permutations stratified by Stand.*

| Within-Stand PERMANOVA model: dist ~ PropBirch + PropSpruce + Height_cm + RegenerationYear; 9999 permutations; permutations stratified by Stand. | | | | | |
| --- | --- | --- | --- | --- | --- |
| Predictor | **Df** | **Sum Of Squares** | **R²** | **F** | **P Value** |
| Propbirch | 1 | 0.645 | 0.01661 | 3.2531 | 0.0202 * |
| Propspruce | 1 | 0.645 | 0.01662 | 3.2535 | 0.7233 |
| Regenerationyear | 1 | 0.540 | 0.01391 | 2.7232 | 0.7150 |
| Height_cm | 1 | 1.185 | 0.03053 | 5.9775 | 0.0001 *** |
| Residual | 178 | 35.291 | 0.90900 |  |  |
| **Total** | 182 | 38.824 | 1.00000 |  |  |

## **Section 6: Differential abundance**

*Figure S8. ANCOM‑BC2 genus‑level differential abundance results comparing planted and naturally regenerated seedlings. Shown are all genera significant at q < 0.05, coloured by trophic mode, before applying the pseudo‑count sensitivity filter. Positive log‑fold changes indicate genera enriched in planted stands, while negative values reflect higher abundance in naturally regenerated stands.*

*Figure S9. ANCOM‑BC2 genus‑level differential abundance results comparing Southwest Finland and Southeast Finland. Shown are all genera significant at q < 0.05, coloured by trophic mode, before applying the pseudo‑count sensitivity filter. Positive log‑fold changes indicate genera enriched in Southeast Finland while negative values reflect higher abundance in Southwest Finland.*

## **Section 7: Results with rarefied data**

*Figure S10. Rarefaction curves for operational taxonomic unit (OTU) richness. Each curve represents an individual sample, illustrating the relationship between sequencing depth (number of reads; x‑axis) and the cumulative number of observed OTUs (y‑axis). Curves show rapid OTU accumulation at low sequencing depths followed by a gradual plateau, indicating approaching sampling saturation. Variation among curves reflects differences in OTU richness and sequencing completeness across samples.*

| Model Shannon: Shannon ~ Area + Origin + NeedleCN + PropSpruce + PropBirch + (1 \| Stand) | | | | |
| --- | --- | --- | --- | --- |
| Fixed Effect | **Estimate** | **Std. Error** | **t value** | **p-value** |
| (Intercept) | 2.545 | 0.306 | 8.33 | 3.16e-13*** |
| Area (SW Finland) | -0.294 | 0.119 | -2.47 | 0.022 * |
| Origin (Planted) | -0.341 | 0.117 | -2.92 | 0.004 ** |
| Needle C:N | 0.017 | 0.008 | 2.21 | 0.029* |
| PropSpruce | -0.890 | 0.450 | -1.97 | 0.051 . |
| PropBirch | -0.450 | 0.199 | -2.26 | 0.027 * |

*Table S10. Coefficient table for rarefied Shannon LMM.*

*Table S11. Coefficient table for rarefied Simpson LMM.*

| Model Simpson: Simpson ~ Area + Origin + NeedleCN + PropSpruce + PropBirch + (1 \| Stand) | | | | |
| --- | --- | --- | --- | --- |
| Fixed Effect | **Estimate** | **Std. Error** | **t value** | **p-value** |
| (Intercept) | 0.791 | 0.076 | 10.47 | < 2e-16 *** |
| Area (SW Finland) | -0.104 | 0.028 | -3.74 | 0.001 ** |
| Origin (Planted) | -0.057 | 0.029 | -1.98 | 0.051 . |
| Needle C:N | 0.004 | 0.002 | 2.37 | 0.019 * |
| PropSpruce | -0.338 | 0.111 | -3.05 | 0.003 ** |
| PropBirch | -0.143 | 0.048 | -2.97 | 0.004 ** |

*Figure S11. Permutational multivariate analysis of variance (PERMANOVA) results for rarefied OTU community composition. Bar plots show the proportion of variance explained (R²) by each predictor for between-stand (left) and within-stand (right) variation in fungal community composition based on Bray–Curtis dissimilarities. Predictors include geographic area, tree height, proportion of birch, proportion of spruce, and regeneration year. Asterisks indicate significance levels (*p < 0.05, **p < 0.01, ***p < 0.001).*

*Table S12.* *Between-stand PERMANOVA results for rarefied fungal OTU community composition based on Bray–Curtis dissimilarities.*

| Between-Stand PERMANOVA model: dist ~ Area + PropBirch + PropSpruce + Height_cm + RegenerationYear; 9999 permutations; by = “margin” | | | | | |
| --- | --- | --- | --- | --- | --- |
| Predictor | **Df** | **Sum Of Squares** | **R²** | **F** | **P Value** |
| Area | 1 | 1.811 | 0.04934 | 10.1973 | 0.0001 *** |
| Propbirch | 1 | 0.681 | 0.01855 | 3.833 | 0.0003 *** |
| Propspruce | 1 | 0.474 | 0.01293 | 2.6715 | 0.0064 ** |
| Height_cm | 1 | 1.043 | 0.02842 | 5.8732 | 0.0001 *** |
| Regenerationyear | 1 | 0.457 | 0.01246 | 2.5761 | 0.0083 ** |
| Residual | 177 | 31.429 | 0.85642 |  |  |
| Total | 182 | 36.689 | 1.00000 |  |  |

*Table S13.* *Within-stand PERMANOVA results for rarefied fungal OTU community composition based on Bray–Curtis dissimilarities.*

| Within-Stand PERMANOVA model: dist ~ PropBirch + PropSpruce + Height_cm + RegenerationYear; 9999 permutations; permutations stratified by Stand. | | | | | |
| --- | --- | --- | --- | --- | --- |
| Predictor | **Df** | **Sum Of Squares** | **R²** | **F** | **P Value** |
| Propbirch | 1 | 0.751 | 0.02045 | 4.0194 | 0.0180 * |
| Propspruce | 1 | 0.614 | 0.01674 | 3.2903 | 0.7900 |
| Regenerationyear | 1 | 0.419 | 0.01141 | 2.2418 | 0.7453 |
| Height_cm | 1 | 1.145 | 0.03119 | 6.1290 | 0.0001 *** |
| Residual | 178 | 33.240 | 0.90576 |  |  |
| **Total** | 182 | 38.698 | 1.00000 |  |  |
