# Supplementary material for: Regional species turnover and local host and forest stand characteristics jointly structure early foliar mycobiomes of Scots pine seedlings
Source: FEMS Microbiol Ecol. 2026 Oct 5;102(11):fiag114. doi: 10.1093/femsec/fiag114 (PMC13673988; doi:10.1093/femsec/fiag114)
Supplement: Supplementary file 1 [file fiag114_supplemental_files.zip › Supplementary methods.docx]

# Supplementary methods: ITS2 sequence processing

**1. Quality assessment and primer removal**

Initial sequence quality profiles were inspected using FastQC and MultiQC. Because ITS primers (ITS3/ITS4) can occur in multiple orientations in raw reads, all four possible orientations (forward, reverse, complement, reverse complement) were generated using the Biostrings package (H. Pagès 2017) in R. Primer removal was performed using Cutadapt (Martin 2011), incorporating orientation-specific FASTA files for both forward and reverse reads. Successful primer removal and correct orientation matching were verified by assessing primer occurrence before and after trimming.

**2. Read filtering**

Filtering was performed using DADA2 with parameters chosen based on quality profiles. Reads containing ambiguous bases (maxN = 0) or exceeding expected error thresholds (maxEE = 2 for both forward and reverse reads) were removed. After primer removal, quality profiles showed high quality scores across their full read lengths. Therefore, no additional truncation or trimming was needed, and all reads were retained at their maximum lengths.

**3. ASV inference and chimera removal**

Error rates were learned separately for forward and reverse reads using up to 2 × 10⁸ bases, with randomization to avoid dominance by highly abundant samples. Reads were dereplicated prior to ASV inference, and the DADA algorithm was used to distinguish true biological sequences from sequencing errors.

Chimeric sequences were removed using the consensus method implemented in DADA2. A stricter threshold (minFoldParentOverAbundance = 2) than the default was applied to reduce the risk of incorrectly removing genuine low abundance ITS variants, which can exhibit high natural variability.

**4. Pipeline validation**

To ensure robustness of parameter choices and pipeline performance, initial analyses were conducted on a subset of samples prior to processing the full dataset. Read retention was monitored across all pipeline stages (input, filtered, denoised, merged, non-chimeric) to identify potential biases or excessive read loss.

**5. Taxonomic assignment and curation**

Primary taxonomic assignment was derived from the UNITE database (Abarenkov *et al.* 2025) and only ASVs assigned to the fungal kingdom were retained. For taxa of primary interest (*Hormonema*, *Alternaria*, *Lophodermium*, *Cyclaneusma*, *Cenangium*, *Fusarium*, *Bisifusarium*, *Gremmeniella*, *Rhizosphaera*, *Gremmenia*, *Botrytis*, *Coleosporium*, and the family Botryosphaeriaceae) additional curation was performed using GeneBank/NCBI (Sayers *et al.* 2011) BLASTn (Zhang *et al.* 2000). For each ASV, the top 5 to 10 BLAST hits were examined for sequence identity, alignment coverage and taxonomic consistency. Species-level identifications were accepted when all high-scoring matches showed ≥99–100% identity to single species, or when discrepancies involved well-established teleomorph-anamorph or heterotypic synonym pairs (e.g. *Sydowia polyspora* / *Hormonema dematioides*).

In some cases, BLAST provided useful clarification at higher taxonomic levels. As an example, for ASV1954, UNITE assigned the sequence to Botryosphaeriaceae, while BLAST suggested *Lasiodiplodia theobromae*. However, comparison with *Diplodia sapinea* reference sequences showed > 98% identity. Given that *D. sapinea* is present in Finland, whereas *L. theobromae* is not, the ASV was assigned to *D. sapinea* as the most biologically and biogeographically plausible taxon. Overall, taxonomic adjustments were minor: in some cases, BLAST allowed refinement to species level, whereas in some genera, namely *Fusarium* and *Alternaria*, we retained a conservative genus-level assignment due to numerous species matching at 99-100% identity.

**6. OTU clustering and filtering**

To reduce the influence of intragenomic ITS variation, ASVs were clustered into OTUs at 97% sequence similarity using the cluster_size algorithm in VSEARCH. This method prioritizes abundant sequences as cluster centroids, thereby reducing the influence of rare sequencing artefacts.

Singletons and low-abundance OTUs (<0.01% relative abundance) were removed to minimize potential noise while retaining ecologically meaningful variation.

## References:

Abarenkov K, Zirk A, Piirmann T *et al.* UNITE general FASTA release for Fungi. UNITE Community, 19 Feb. 2025 [application/gzip]. https://doi.org/10.15156/BIO/3301229.

H. Pagès PA. *Biostrings*. Bioconductor, 2017. https://doi.org/10.18129/B9.BIOC.BIOSTRINGS.

Martin M. Cutadapt removes adapter sequences from high-throughput sequencing reads. *EMBnet j* 2011;**17**(1):10. https://doi.org/10.14806/ej.17.1.200.

Sayers EW, Barrett T, Benson DA *et al.* Database resources of the National Center for Biotechnology Information. *Nucleic Acids Research* 2011;**39**(Database):D38–51. https://doi.org/10.1093/nar/gkq1172.

Zhang Z, Schwartz S, Wagner L *et al.* A Greedy Algorithm for Aligning DNA Sequences. *Journal of Computational Biology* 2000;**7**(1–2):203–14. https://doi.org/10.1089/10665270050081478.
